# Exploring the Intrinsic Structural Plasticity and Conformational Dynamics of Human Beta Coronavirus Spike Glycoproteins

**DOI:** 10.1021/acs.jcim.5c00990

**Published:** 2025-07-17

**Authors:** Yago Ferreira e Silva, Harold Hilarion Fokoue, Paulo Ricardo Batista

**Affiliations:** † Programa de Computação Científica, Vice-Presidência de Educação, Informação e Comunicação, 37903 Fundação Oswaldo Cruz, Av. Brasil 4365, Residência Oficial, Manguinhos, 21040-900 Rio de Janeiro, Brazil; ‡ Programa de Pós-graduação em Biologia Computacional e Sistemas, Instituto Oswaldo Cruz, Fundação Oswaldo Cruz, Av. Brasil 4365, Manguinhos, 21040-900 Rio de Janeiro, Brazil

## Abstract

The spike (S) glycoprotein of human beta coronaviruses (HCoVs) is central to viral entry, receptor engagement, and immune evasion. Here, we present an in-depth computational analysis of spike conformational dynamics across HCoVs, with a focus on SARS-CoV-2 and its variants. Leveraging a large cryo-EM structural ensemble and integrative modeling approaches, we dissect the intrinsic plasticity and variant-specific motions of the spike protein. Our results show that, despite substantial sequence divergence, HCoV spikes retain the ability to sample open and closed receptor-binding domain (RBD) states. For SARS-CoV-2, a hinge-like RBD opening motion dominates the conformational landscape, modulating ACE2 accessibility. Ensemble and single-structure normal modes revealed conserved dynamic domains and hinge regions and showed strong agreement with experimental structural transitions. Ligand binding rather than the D614G mutation was the principal driver of RBD opening, with multiple open RBDs observed predominantly in ligand-bound states. Notably, Omicron spike structures favored closed RBDs in the *apo* form but remained capable of ligand-induced opening. Dynamical network analysis identified an Omicron-specific remodeling of interdomain communication, altering the mechanical connectivity between RBD, NTD, and S2 subunits. Analysis of single-experiment multimodel cryo-EM data from the Beta variant captured temperature-dependent metastable states, validating ensemble-based modeling. Finally, hybrid molecular dynamics simulations successfully reproduced the spike experimentally observed in conformational space, unlike standard MD. These findings offer mechanistic insight into spike conformational dynamics, supporting the design of variant-adapted therapeutics and vaccines.

## Graphical abstract

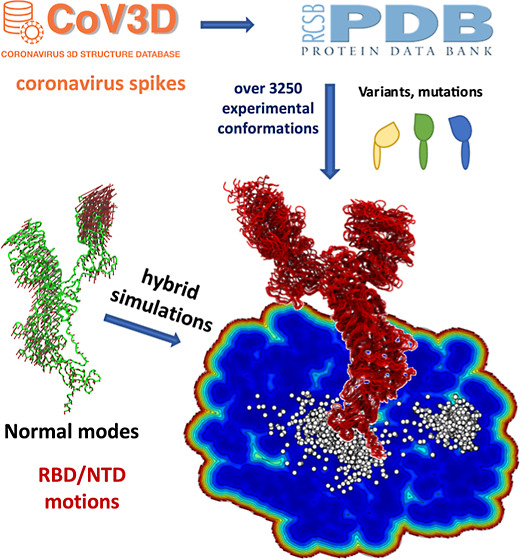

## 1 Introduction

The severe acute respiratory syndrome coronavirus 2 (SARS-CoV-2), first identified in late 2019, is the causative agent of the ongoing COVID-19 pandemic.^1^ As a member of the family, SARS-CoV-2 shares similarities with other human beta coronaviruses (HCoVs) responsible for significant diseases, such as SARS-CoV and MERS-CoV.^2^ SARSCoV-2 has a positive-sense single-stranded RNA genome that encodes four structural proteins: nucleocapsid, envelope, membrane, and spike, along with 16 nonstructural proteins and nine accessory proteins.^3^ These proteins contribute to the virus’s ability to replicate, assemble, and evade the host’s immune system.

The spike (S) protein is crucial for the virus’s entry into host cells. It protrudes from the viral surface in its prefusion state and binds to the angiotensin-converting enzyme 2 (ACE2) receptor, which is expressed on cells of the respiratory tract.^4^ Upon receptor binding, the spike undergoes conformational changes that trigger the fusion of the viral and host cell membranes, allowing the viral RNA to enter the host cell. The S protein is also a major target of the immune response, making it the focal point for the development of vaccines and therapeutics.^5^ Structurally, the S protein is a homotrimer, with each protomer consisting of two subunits: S1, responsible for receptor binding, and S2, which mediates membrane fusion.^6^ The S1 subunit contains the N-terminal domain (NTD), the receptor-binding domain (RBD), and two subdomains (C1 and C2) (Figure S1). The RBD is critical because it directly interacts with ACE2 through a specific region called the receptor-binding motif (RBM). Understanding the dynamics of this interaction has been the subject of intense study, given its importance for viral entry.^7^ The S protein’s RBD fluctuates between closed (down) and open (up) states. In the open conformation, the RBD is exposed and ready to engage ACE2, while the closed state conceals the RBD, preventing receptor interaction and minimizing immune detection.^8^ Cryo-electron microscopy (cryo-EM) studies have shown that the spike trimer can bind up to three ACE2 receptors, with each protomer capable of binding one ACE2 molecule when the RBD is in its open state.^9^

Early in the pandemic, the S protein of the Wuhan-1 reference strain (hereafter referred to as “original”) acquired the D614G mutation, which shifted the equilibrium toward a more open RBD conformation, increasing the virus’s transmissibility.^10,11^ This mutation allowed greater structural stability in a region known as the 630 loop (residues 620–640), enhancing viral fitness and enabling the rapid acquisition of additional mutations.^12,13^ As a result, the D614G mutation has become a hallmark of many subsequent SARS-CoV-2 variants. Several notable previously circulating variants of concern have emerged, each distinguished by key mutations in the S protein. These include: Alpha (B.1.1.7), first detected in the UK, which increased transmissibility and mortality;^14^ Beta (B.1.351), first identified in South Africa, featuring enhanced immune evasion due to shared mutations with Alpha;^15^ Gamma (P.1), first detected in Brazil, which introduced 17 unique mutations in the S protein and shared mutations with Alpha and Beta;^16^ Delta (B.1.617.2), first identified in India, which rapidly became the dominant variant globally due to its high transmission rate;^17^ and Omicron (B.1.1.529), identified in November 2021 in South Africa, which rapidly replaced Delta due to its high number of mutations, particularly in the S protein, including more than 30 mutations, as well as three deletions and one insertion.^18^ Since February 2022, the SARS-CoV-2 Omicron variant has accounted for over 98% of the publicly available sequences. Currently, these earlier variants (Alpha, Beta, Gamma, Delta) are no longer classified as variants of interest or variants under monitoring, as the focus has shifted to newer Omicron sublineages.^19^

The Omicron variant mutations, particularly N501Y, T478 K, and K417N, have significantly increased its ability to evade the immune response, enhancing both transmissibility and immune escape.^20–22^ The variant includes several sublineages, such as BA.1, BA.2, BA.4, BA.5, and the recombinant strain XBB, each of which carries distinct mutations that affect viral fitness, binding affinity to ACE2, and immune evasion.^23^ For instance, BA.1 exhibited 39 mutations (15 in the RBD), while BA.2 and BA.5 showed enhanced transmissibility despite having fewer mutations in the spike protein. XBB and its subvariants, such as XBB.1 and XBB.1.5, exhibit superior immune evasion compared to earlier Omicron sublineages like BA.5, making them particularly concerning in terms of their potential to cause breakthrough infections.^24,25^

Despite significant progress in solving structures of spike variants, structural data alone cannot fully explain the dynamics of S protein conformational changes, particularly the transition between closed and open RBD states. Given that S protein dynamics is crucial to its function, molecular dynamics (MD) simulations offer valuable insights into its flexibility, residue interactions, and how mutations alter its behavior. Recent MD simulations have provided insights into the structural flexibility of the S protein, revealing allosteric sites and epitopes that may serve as druggable targets.^26–40^ These studies have been pivotal in elucidating how mutations like D614G and those found in the Omicron variant affect the S protein’s ability to mediate viral entry. Single-molecule experiments showed that Omicron RBD frequently adopts a closed conformation, reducing exposure to neutralizing antibodies, and compensates for fewer ACE2 binding interactions by maintaining longer attachment times, which enhances immune evasion and viral attachment.^41^ However, understanding the S protein’s conformational dynamics remains a challenge.

A key question in SARS-CoV-2 biology is how the spike protein’s conformational dynamics enable adaptability across variants despite extensive sequence variation. This study addresses that by integrating large-scale structural data with advanced conformational analysis to examine how plasticity is influenced by mutations, ligand binding, and temperature. We explore whether distinct dynamic profiles, such as those in Omicron and beta, represent adaptive mechanisms balancing immune evasion and receptor engagement. Focusing on HCoVs, we assess spike flexibility, its impact on ligand affinity, and the conformational landscape of the SARS-CoV-2 variants. Using single-structure and ensemble normal-mode analysis, along with hybrid MD simulations, we characterize the dynamic behavior of key variants. Our findings highlight the unique conformational features of the Omicron and offer new insights into the structure–function relationship of spike dynamics, with implications for viral transmission and immune escape.

## 2 Materials and Methods

### 2.1 Experimental Structural Ensemble of the Spike Proteins

To study the plasticity of the human beta coronaviruses, the spike experimental structures were retrieved, classified, and analyzed as follows (see the experimental workflow in Figure S2). The scripts are available as Supporting Information (https://github.com/yago52/tutorial_spike).

#### 2.1.1 Ensemble Building

The ensemble of protomeric structures for the full SARS-CoV-2 spike protein (head fragment) was generated using the *buildPDBEnsemble* function from ProDy^42^ (version 2.3). Each chain was processed individually, with the 6VXX structure (chain A) serving as a reference. A sequence identity cutoff of 90% and a minimum sequence overlap of 50% were applied, followed by gap-based filtering to retain structures with at least 90% sequence coverage. The individual chain ensembles were subsequently merged into a final ensemble, comprising a total of 2.872 structures. For other human beta coronaviruses, the same script and reference structure were used, but a sequence identity cutoff of 20% was applied instead.

We also created a new ensemble comprising single-experiment multimodel cryo-EM structures corresponding to the Beta variant (B.1.351). All 20 models from each PDB entry were included, resulting in a total of 120 conformations (considering all protomers), derived from samples equilibrated during vitrification at two distinct temperatures: 4 (9GDX) and 37 °C (9GDY).

#### 2.1.2 Classification of Ensemble Structures

To analyze the structural diversity of the spike protein, two ensembles of experimental structures were created: one comprising non-SARS-CoV-2 human beta coronaviruses and another containing only SARS-CoV-2 structures. A list of PDB-IDs for coronavirus spike proteins from SARS-CoV, SARS-CoV-2, and MERS-CoV was obtained from Cov3d, a specialized database that is updated weekly.^43^ Alternatively, the PDB-IDs for OC43 and HKU1 were directly obtained from the Protein data bank (PDB)^44^ and UniProt^45^ databases.

Cov3d provides a series of tables with filtering options, including *(i)* whether the spike protein is complexed with a ligand (specifying ligand name, type, and associated chain(s)); *(ii)* whether the structure represents a full-length protein or a fragment; and *(iii)* SARS-CoV-2 classification by variant. The tables were processed with the *Pandas* package^46^ (version 2.1.1) in Python (version 3.9).

##### 2.1.2.1 *Apo* and Ligand-Bound Structures

The ensemble structures were classified based on the ligand using the data from the Cov3d. Such information was related to each PDB-ID, but within the trimeric bound structures, we evaluate whether each protomer has interactions with the ligands by calculating the number of contacts; if the protomer has no contact with the ligand, it is classified as an *apo* protomer. The antibodies were classified in classes according to domain targeting.

##### 2.1.2.2 Mutation on 614th Residue

The residue at position 614 was identified for all of the complete structures in the ensemble using ProDy. The data set was subdivided into three primary groups: 614D, 614G, and 614­(C, N, T, Y, A, L, P, E, and I).

##### 2.1.2.3 SARS-CoV-2 Variant Classification

The initial variant classification for the SARS-CoV-2 structures was obtained from the Cov3d database. However, approximately 45% of the PDB entries in the ensemble were not classified by Cov3d. To ensure comprehensive classification, we performed a sequence-based analysis of the entire ensemble. Complete .fasta sequences of all spike protein structures were retrieved from the PDB server, and a multiple sequence alignment (MSA) was performed using MUSCLE^47^ with a Biopython^48^ script. Short segments at the N- and C-terminal ends were truncated (residues 1–13 and >1142, UniProt code P0DTC2). Based on the MSA, a sequence distance matrix was constructed using the *buildSeqidMatrix* function from ProDy, and a phylogenetic tree was generated with the *calcTree* function, applying the weighted algorithm. Clustering was performed using the *scipy.cluster.hierarchy.dendrogram* function from the *SciPy* package.^49^ Clusters consistently contained either a single variant type previously classified by Cov3d or unclassified structures. For clusters with single classifications, all members were labeled with the same variant as the preclassified entries. In clusters where no Cov3d-classified entries were present, variant information was manually verified through PDB annotations and the corresponding literature. This comprehensive approach enabled consistent classification across the entire ensemble.

### 2.2 Prediction of Intrinsic Motions of SARS-CoV-2 Spike

#### 2.2.1 Single Structure Normal-Mode Analysis

NMA was used to probe the intrinsic flexibility of the representative spike conformers. By computing vibrational modes from the elastic network of individual structures, it reveals the built-in potential for large-scale motion encoded by the spike topology and contacts. The 20 slowest nontrivial normal modes were calculated with the ProDy package using the anisotropic network model (ANM) for the ensemble reference structure (6VXX_A). First, the Hessian matrix was built (*buildHessian* method) based on the Cα Cartesian atomic coordinates, using a 15 Å pairwise-interactions cutoff and a gamma spring constant of 1 N/m; and the modes were calculated by diagonalizing this matrix (*calcModes* method).

#### 2.2.2 Ensemble Normal Modes

The ensemble normal modes integrate structural diversity and highlight conserved, statistically dominant dynamic features across different conformational states and variants. They were calculated using the *calcEnsembleENMs* function in the ProDy package using the Gaussian network model (GNM) method. The Kirchhoff matrix was constructed with a 10 Å cutoff for the pairwise atomic interactions and a spring constant of 1 N/m. For each ensemble member, the 20 slowest nontrivial normal modes were computed. The modes calculated for each structure (referred to as modesets) were automatically matched to a modeset and ordered accordingly to the reference structure modes.

### 2.3 Ensemble Analysis

#### 2.3.1 Principal Component Analysis (PCA)

PCA was applied to the SARS-CoV-2 experimental cryo-EM structural ensemble to extract the dominant motions sampled across spike variants. This data-driven method captures the observed conformational diversity by reducing the dimensionality of the structural data set without relying on predefined force fields or energy models. PCA was performed over the Cα-atom Cartesian atomic coordinates using the ProDy package. The covariance matrix was built (*buildCovariance* method), and the PCs were calculated by diagonalizing this matrix (*calcModes* method).

#### 2.3.2 RBD Conformation Classification

PC1 projection was used to classify RBD conformations: protomers with values >5 as open; and ≤5 as closed states. This threshold was chosen based on the natural inflection point between these two populations, supported by the RMSD and RBD angle correlations.

#### 2.3.3 Global Structural Measurements and Collective Variables (CVs) Used To Describe Spike Flexibility

##### 2.3.3.1 Root Mean Square Deviation (RMSD)

The spike alpha carbon RMSD was calculated based on the reference structure using the ProDy function *calcRMSD*.

##### 2.3.3.2 Radius of Gyration Calculation (Rg)

The *Rg* of each structure was calculated by using the ProDy *calcGyradius* function.

##### 2.3.3.3 NTD/RBD Interdomain Distance

The Euclidean interdomain distance between the NTD (residues 27–303) and the RBD (residues 319–541) centers of mass (CoMs) was computed using a custom Python script based on the ProDy toolkit.

##### 2.3.3.4 Distances between the RBDs or NTDs within the Trimmer

As described in ref 50, six collective variables (CVs) were defined to represent distances between the CoMs of the RBDs and NTDs within the spike trimer. CV1–CV3 correspond to RBD–RBD distances between chains 1–2, 2–3, and 3–1, respectively; CV4–CV6 analogously represent NTD–NTD distances between the same chain pairs (Figure S3A).

##### 2.3.3.5 Area of the Triangle Formed by RBDs or NTDs within the Spike Trimmers

To quantify the spatial arrangement of RBDs or NTDs within each spike trimer, we calculated the area of the triangle defined by the CoM of each RBD or NTD within the spike trimmer for each structure in the ensemble using Heron’s formula, as follows: $S_{RBD}=\sqrt{s\left(s-CV1\right)\left(s-CV2\right)\left(s-CV3\right)}$ , where *s* is the triangle semiperimeter: *s* = (CV1 + CV2 + CV3)/2. Similarly, for the area of the triangle formed by each NTD (*S*_NTD_), the CVs 4, 5, and 6 were used instead of CVs 1, 2, and 3.

##### 2.3.3.6 RBD Angle (Residues 405, 620, and 991)

This angle has been shown to reflect ACE2 accessibility and track RBD conformational transitions.^39^ Due to the absence of residue 622 in our structural ensemble, we used the angle formed by residues 405, 620, and 991 as a representative metric (Figure S3B).

##### 2.3.3.7 Vector-Based Analysis

A robust CV proposed by ref 51 was shown to efficiently capture spike conformational changes. This CV is built using the CoMs from 9 structurally relevant regions of the spike protein: NTD (27:43 and 54:271), NTD-β (116:129 and 169:172), NTD′ (44:53 and 272:293), RBD (330:443 and 503:528), RBD-α (403:410), SD1 (323:329 and 529:590), SD2 (294:322 and 591:696), S2-β (717:727 and 1047:1071), and CD (711:716 and 1072:1122), according to Figure S3C.

#### 2.3.4 Number of Contacts between Protomers and Ligands

Using ligand information from Cov3D, a Python script employing the ProDy and Pandas libraries was developed to analyze the structural ensemble. For each structure, when a ligand was detected, the corresponding chain was identified, and the number of heavy atoms from each spike protein residue located within 4 Å of any ligand heavy atom was computed.

#### 2.3.5 Dynamical Domain Analysis and Hinge Point Detection

The dynamical domains were classified employing the *DynDom* ^52^ program, which analyzes structural transitions between two conformers. The normal-mode-displaced structures and the two experimental structures were uploaded and analyzed in the server. The hinge points associated with the conformational changes were also predicted using the *DynDom*.

#### 2.3.6 Clustering Analyses

The SARS-CoV-2 spike ensemble structures were clustered using the *g_cluster* module of the GROMACS package,^53^ with the *gromos* algorithm and a 1.5 Å RMSD cutoff.

#### 2.3.7 Overlap and Subspace Overlap between Modes and PCs

The ProDy package was used to calculate the overlap (*CalcOverlap* function) between the two sets of modes. The first set corresponds to the first 5 PCs from the SARS-CoV-2 experimental ensemble, while the second corresponds to the 5 slowest nontrivial modes of the reference structure. The modes were normalized prior to overlap calculation. A graphical table was done directly using the function *showOverlapTable.* The subspace overlap was calculated (*calcSubspaceOverlap* function) between each PC and the subspace spanned by the first five normal modes.

#### 2.3.8 Ensemble Spectral Overlap

The spectral overlap was calculated to compare the dynamics of the individual ensemble members using the function *calcEnsembleSpectralOverlaps*, according to the formula: $s\left(A,B\right)=1-\frac{d\left(A,B\right)}{\sqrt{trA+trB}}$ ,^54^ where *s* is the spectral overlap between NM (or a set of modes) of conformation *A* and *B*, *d* is the difference in the covariance of *A* and *B* matrices, and tr denotes the trace of the corresponding matrix. The arccosine of this value provides a distance metric, allowing for the construction of a spectral distance matrix by computing the overlap for all pairs in a mode ensemble. This matrix can then be used to generate a dynamics-based “phylogenetic” tree.

#### 2.3.9 Signature Dynamics Analysis

##### 2.3.9.1 Signature Profiles

The dynamic signatures of the first three slowest nontrivial ensemble normal modes were calculated individually and collectively using the *showSignatureMode* and their corresponding cross-correlation matrix with *showSignatureCrossCorr* ProDy functions.

##### 2.3.9.2 Comparing Ensemble Sequence, Dynamics, and Structure

Three matrices were calculated: *i.* pairwise RMSD, using ProDy ensemble’s method *getRMSDs* (*pairwise* = *True*); *ii.* spectral overlap distance (20 nontrivial slowest mods) *calcEnsembleSpectralOverlaps* (*distance* = *True*); *iii.* sequence distance (*seqdist_matrix*), generated from the sequence identity matrix (*seqdist_matrix* = 1 – *seqid_matrix*), which was constructed from the ensemble MSA.

#### 2.3.10 Dynamical Network Analysis

##### 2.3.10.1 Network Generation from Ensemble Normal-Mode Analysis Data

A consistent structural ensemble was generated from the ProDy-derived SARS-CoV-2 models (Section 2.1.1) using the *pdbaln* function from the Bio3D package,^55^ which was used for all analysis of this section. The 20 slowest nontrivial normal modes for each structure were calculated using the *nma* function with the HCA model.^56^ These modes were used to calculate the dynamic cross-correlation matrix (DCCM) via the *dccm* function, where each element (*C*_*ij*_) represents the degree of dynamic coupling between residues *i* and *j*. A value of *C*_*ij*_ = 1 indicates that the fluctuations of residues *i* and *j* are completely correlated; *C*_*ij*_ = −1, is completely anticorrelated; and *C*_*ij*_ = 0 is not correlated. Filters on the DCCM (*filter.dccm* function) were applied based on groups: RBD conformation (closed and open); ligands (*apo*, antibodies, receptors); 614th residue (614D, 614G, others); and SARS-CoV-2 variant (Omicron and non-Omicron). The correlation threshold was set to 0.3 (*cutoff.cij* = *0.3*), and all residue pairs, including neighboring residues (scut = 0), were retained.

##### 2.3.10.2 Protein Dynamic Correlation Network Construction and Community Analysis

Network construction. Correlation network analysis (CNA) was performed using the Bio3D *cna* function using the random walk clustering method. The filtered DCCMs were used as input for a residue-based network community organization detection.

Network refinement. To identify equivalent communities among the groups, networks were refined using the *remodel.cna* function. Residue pairs involving four sequential neighbors were excluded (scut = 4). Nodes were defined by spike protein domains and interdomain regions via the member parameter. The input correlation matrix was defined as the sum of all intercommunity numeric square matrices (*C*_*ij*_ *Sum*), containing absolute values from the atomic correlation matrix for each community (*cij.community*), using method = “sum”. Edge colors reflected the normalized dynamic correlation between groups (*col.edge* = “*feature*”), with a correlation threshold of 0.25.

PCA of an array of DCCMs from the ensemble normal modes. PCA was performed using the *pca.array* function on a series of DCCMs, each derived from normal modes calculated for all ensemble structures. The function returns *M* eigenvalues and eigenvectors, where *M* is the number of input matrices, and each eigenvector has a dimension of *N*(*N* – 1)/2, with *N* being the number of matrix rows/columns.

### 2.4 Standard MD Simulation Trajectories

Two 10 μs MD simulation trajectories of the trimeric structure of the SARS-CoV-2 S protein were carried out by David Shaw’s group and deposited under the codes DESRES-ANTON-11021566 and DESRES-ANTON-11021571 and were obtained from the site (https://covid.molssi.org/org-contributions/). These simulations were performed with the AMBER program^57^ using Amber ff99SB-ILDN and *Glycam* force fields. TIP3P water models were used in all of the simulations. The first simulation was started with the S protein in the closed state (PDB 6VXX);^9^ and the second, from a partially open state (PDB 6VYB).^9^ The C and N termini were completed with amide and acetyl groups, respectively. Counterions were inserted to neutralize the system, reaching a 0.15 M NaCl concentration. The simulations were maintained at 310 K using the *NPT* ensemble.

### 2.5 MDeNM Simulations and Conformational Free Energy Estimation

#### 2.5.1 System Preparation, Minimization, and Equilibration

The initial structures of the fully glycosylated spike protein head-only models (residue 1–1146) for the 6VSB structure were taken from the COVID-19 Proteins Library of the CHARMM-GUI Archive (https://www.charmm-gui.org/?doc=archive&lib=covid19). Preparation steps were conducted with the CHARMM-GUI Web server. Each system was placed in a cubic box with a 14 Å layer of TIP3P water molecules. Counterions were inserted to neutralize the system, reaching a 0.15 M NaCl concentration. Then, an energy-minimization protocol was performed, starting with the conjugate-gradient algorithm, keeping protein heavy atoms harmonically restrained with a force constant of 50 kcal mol^–1^ Å^–2^ to avoid structural distortions. The following steps using the same algorithm were carried out using decreasing force constants (up to 2.5 kcal mol^–1^ Å^–2^). Then, the atomic velocities were assigned accordingly to a Maxwell–Boltzmann distribution corresponding to 50 K and then slowly increased to 300 K during a 1 ns heating MD, using a 1 fs integration time. In the equilibration step, the positional restraints were gradually decreased to zero during the first half of a 3 ns constant temperature MD, while in the remaining part all restraints were removed.

#### 2.5.2 MDeNM Simulations

The MD with excited normal modes (MDeNM)^58^ simulations were performed using the MDexciteR tool (https://github.com/mcosta27/MDexciteR).^59^ This tool is based on the combination of R internal libraries and functions from the Bio3D package, allowing users to generate and process files to run MDeNM with an external MD software, the NAMD v.2.3.^60^ MDeNM simulations were performed in the NPT in explicit solvent using periodic boundary conditions. van der Waals interactions were calculated up to 10 Å, being approximated until 12 Å by using a switching function. Electrostatic interactions were treated with the PME algorithm using a 10 Å cutoff. The SETTLE and SHAKE algorithms were used during MD simulations to fix bonds involving hydrogen atoms in water molecules and protein. Pressure was kept constant at 1 atm during equilibration and production using the Langevin piston method. In these steps, the temperature was also kept constant at 300 K using the Langevin thermostat with a damping coefficient of 1 ps^–1^. Each of 400 replicates corresponds to 20 cycles of excitations (linear combinations of the first 3 nontrivial normal modes) for a time of 2 ps (totaling 40 ps per replicate), with an excitation temperature of 2 K. Additionally, the structures, configuration files, and input scripts required to perform the MDeNM simulations are provided and can be accessed directly at: https://github.com/yago52/tutorial_spike/blob/main/mdenm_spike.tar.gz.

#### 2.5.3 Free Energy Landscape Analysis

The free energy landscape (FEL) calculation protocol involved clustering the concatenated structures generated by MDexciteR, followed by relaxation MD simulations initiated from each cluster centroid. The GROMOS clustering algorithm was used with an RMSD cutoff of 1 Å to identify representative conformations. Each of the 137 centroid structures was subjected to a 1 ns unrestrained production MD simulation, with trajectory frames recorded at 2 ps intervals. These trajectories were concatenated into a single file for subsequent analysis.

FELs were computed by using a custom R script. The free energy difference (Δ*G*α) of a given state α relative to the most populated reference state was calculated accordingly

1 $$G_{\alpha }=-k_{B}T\ln\left[\frac{P\left(q_{\alpha }\right)}{P_{\max}\left(q\right)}\right]$$

where *k*_B_ is the Boltzmann constant, *T* is the temperature of the simulations (300 K), and *P*(*q*_α_) is an estimate of the probability density function obtained from bidimensional kernel density estimates of projections onto NM vectors. *P*_max_(*q*) corresponds to the probability of the most frequently visited state.

### 2.6 Figures and Movies

All figures and movies were generated using VMD^61^ (version 1.9.4a51), the open-source version of PyMol;^62^ and the python plots (matplotlib,^63,64^ seaborn^64^ and ProDy tools); and R (4.4.1) plots using the base graphics functions and Bio3D tools.

## 3 Results and Discussion

### 3.1 Human Beta Coronaviruses Share Similar RBD Plasticity

HCoVs, including SARS-CoV, SARS-CoV-2, MERS-CoV, OC43, and HKU1, show significant variations in their spike protein sequences, primarily within the S1 subunit. Notably, the overall spike sequences of SARS-CoV and SARS-CoV-2 share approximately 76% identity, while their identity with MERS-CoV and other endemic HCoVs (OC43 and HKU1) is lower, around 30–40%.^65,66^ To investigate how sequence variations influence the structural plasticity of the spike protein across HCoVs, we constructed an ensemble of all of the experimentally available 3D structures. This ensemble comprises 2872 SARS-CoV-2 conformations, 114 of SARS-CoV, 78 of MERS-CoV, 45 of HKU1, and 39 of OC43. A detailed list of these PDB-IDs, along with relevant annotations, is provided in Table S1.

PCA of Cα-atom Cartesian coordinates showed that all HCoV spike proteins, except OC43, sampled both closed and open RBD conformations, highlighting their shared functional flexibility (Figure 1A). Although OC43 structures were only experimentally observed in the closed state, the limited availability of full-length OC43 spike structures may hinder its experimental observation.

**Figure 1:**
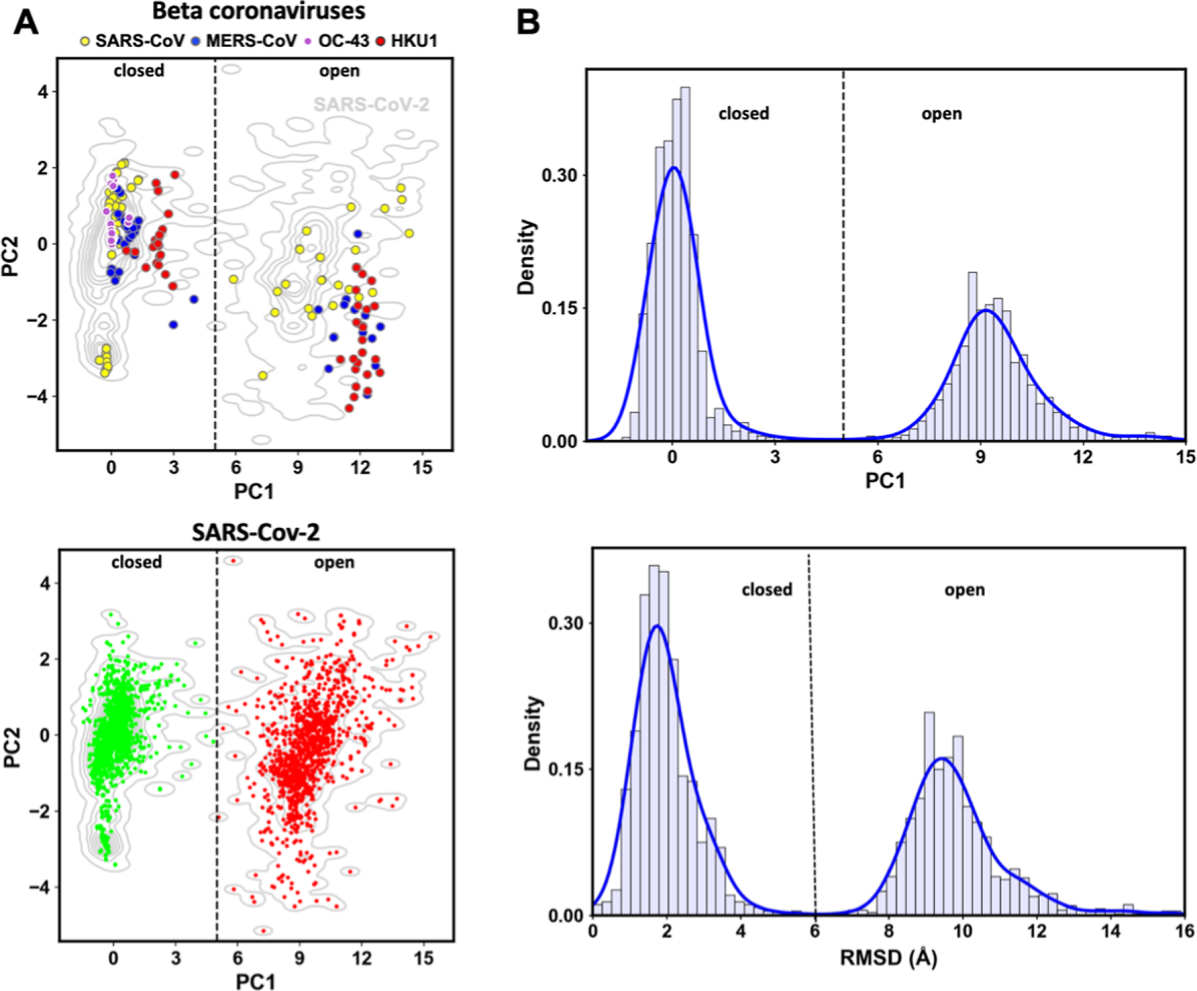
RBD plasticity of human beta coronaviruses. (A) PC projections of spike protomeric structures from HCoVs onto PC1 and PC2. (top) SARS-CoV (yellow), MERS-CoV (blue), OC43 (purple), and HKU1 (red); (bottom) SARS-CoV-2. Light gray contour lines represent the SARS-CoV-2 density distribution. A dashed line marks the boundary between closed (green) and open (red) states. (B) Distribution of PC1 projections (top); RMSD distribution showing two distinct conformational populations (bottom); the dashed lines separate open and closed RBD conformations.

Moreover, recent evidence suggests OC43 can transition to an open state, exposing the S1B domain (RBD equivalent), as indicated by neutralizing antibodies targeting cryptic S1B epitopes.^67^ Unlike SARS-CoV-2, both OC43 and HKU1 belong to the subgenus. For HKU1, spike opening is not spontaneous but is instead triggered by sialoglycan binding, raising the possibility of a similar ligand-dependent regulatory mechanism in OC43.^68^

### 3.2 NTD/RBD Motions Govern SARS-CoV-2 Experimental Dynamics

The SARS-CoV-2 structural ensemble consists of 2872 conformations derived from 968 unique PDB entries. The maximum structural variation within the ensemble reaches an RMSD of 17.5 Å, compared to the reference structure (Figure S4A). Sorting the ensemble by RMSD reveals two distinct populations (Figure S4B and Movie S1). RMSD density plots confirm this separation: a major population (57.9%, 1663 structures) with the RBD in the closed conformation and a second population with the RBD in the open state (Figure 1B).

PCA performed over the SARS-CoV-2 ensemble Cartesian coordinates identified the primary motion (PC1), which resembles a hinge or pivoting movement where the RBD rotates outward to expose the ACE2-binding surface (Figure 2 and Movie S2). This transition occurs around specific hinge regions near the central β-sheet core of the RBD and its connection to subdomain C1 of the S1 subunit. PC1 accounts for more than 84% of the total variance (Figure S5B) and effectively distinguishes between the open and closed RBD states (Figure 1). An open RBD conformation is defined as a PC1 projection greater than 5 Å (Figure 1).

**Figure 2:**
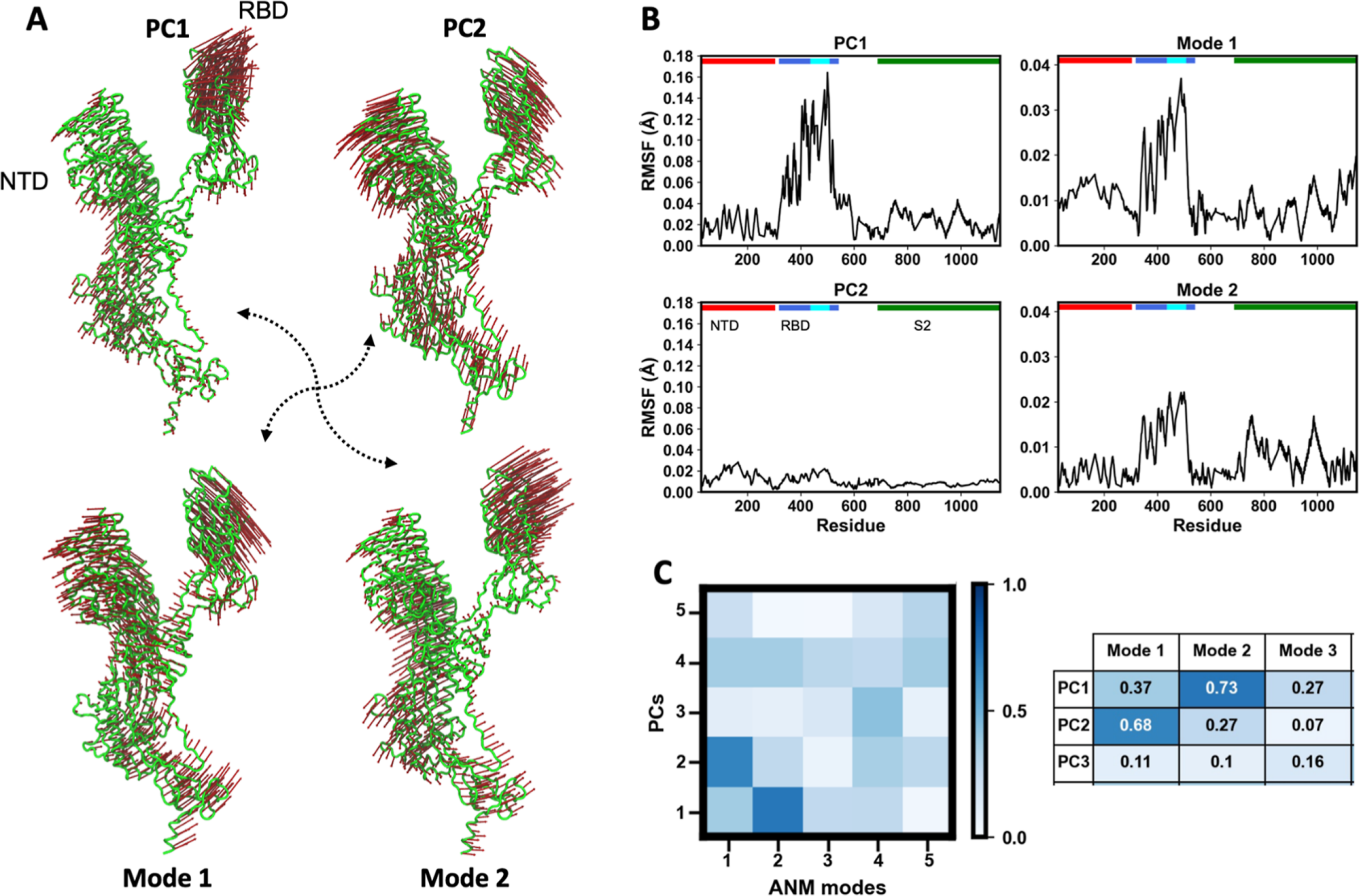
SARS-CoV-2 spike intrinsic motions: close correspondence between experiments and normal modes. (A) Directions of the first two normal modes (bottom) and experimental PCs (top), represented by red arrows (scaled to 5 Å RMSD) on the spike structure shown as a green tube. (B) RMSF profiles for PCs (left) and normal modes (right), with colored bars indicating spike structural domains: NTD (red), RBD (royal blue), RBM (cyan), and S2 (green). (C) Overlap between the first five PCs and normal modes, shown as a blue-shaded matrix; a zoomed-in version with numeric values is displayed on the right.

The similarity between the RMSD and PC1 density plots (Figure 1B) indicates that the primary PC effectively captures the dominant global conformational variations reflected by the RMSD. Further analysis revealed strong linear correlations between PC1 and several structural descriptors: RMSD (*R* = 0.99), spike radius of gyration (*R* = 0.90), and the interdomain distance between the NTD and RBD (*R* = 0.86) (Figure S6). These findings underscore the central role of PC1 in describing the principal conformational transitions of the spike protein, particularly a hinge-like motion of the RBD that modulates exposure of the ACE2 binding site by changing its proximity to the NTD. In contrast, PC2 primarily captures an interdomain opening/closing motion between the NTD and RBD, as illustrated in Figure 2 and Movie S3. Negative PC2 values correspond to shorter interdomain distances. However, when considering the full ensemble, PC2 exhibits only a weak correlation with the NTD-RBD interdomain distance (*R* = 0.40) (Figure S6B). Interestingly, when the ensemble is separated into open and closed RBD conformations, PC2 shows a strong correlation within each subgroup (*R* = 0.81 and 0.82, respectively). This suggests that while PC2 does capture relevant interdomain dynamics, its relationship with structural metrics becomes more evident when analyzing distinct conformational states individually. In other words, this intrinsic motion occurs within both RBD states independently of the broader conformational transition between them, which is primarily described by PC1. Together, PC1 and PC2 account for over 90% of the total conformational variability in the ensemble and thus define an essential subspace for describing the experimental dynamics of the SARS-CoV-2 spike protein.

A computational study previously defined an RBD angle formed by residues 405, 620, and 991 (Figure S3B) as a geometric descriptor capable of distinguishing ACE2 accessibility and monitoring the RBD conformational transition pathway.^39^ Based on predicted conformational pathways, the authors proposed two RBD angle ranges: an ACE2-inaccessible range (31.6° to 52.2°) and an ACE2-accessible range (52.2°–84.8°).

In contrast, our analysis is based on an ensemble of experimentally determined SARS-CoV-2 spike structures. We calculated the RBD angle across this ensemble and observed that its density distribution closely resembles that of PC1 (Figure S4D), clearly separating open and closed states into two distinct populations. Importantly, a strong linear correlation was observed between the RBD angle and PC1 (*R* = 0.99) (Figure S4C), further validating the RBD angle as a robust descriptor of spike conformational dynamics.

To empirically assess ACE2 accessibility, we analyzed the RBD angle distribution in spike structures experimentally resolved in complex with ACE2. These structures exhibited RBD angles ranging from 49.7° to 74.7°, with a density distribution concentrated between approximately 55° and 72°. This experimentally derived range aligns well with the previously predicted ACE2-accessible interval and supports the use of the RBD angle as a proxy for the ACE2 binding potential. Notably, this range excludes the mink spike variant (Y453F), where the mink ACE2 is bound to a spike in a closed conformation (PDB: 8T22) with an RBD angle of 34.8°, falling within the computationally defined inaccessible region. Altogether, these findings reinforce the conclusion that binding of ACE2 preferentially stabilizes the open RBD conformation of the SARS-CoV-2 spike protein.

Notably, PC1, the RBD angle, and the interdomain NTD–RBD distance are strongly correlated and capture the same essential RBD opening motion. For interpretability and consistency, we prioritized RBD angle and PC1 as principal descriptors in our biological analyses, using RMSD and interdomain distance only as supporting metrics when relevant. These combined projections define a conformational subspace that efficiently describes the spike’s functional transition between receptor-inaccessible and receptor-accessible states.

A vector-based analysis considering the CoMs of nine specific spike structural domains (Figure S3C) was recently proposed as a CV to describe spike flexibility.^51^ Based on this CV, we generated a PDB file for each structure in the ensemble, containing the Cartesian coordinates of the CoMs of the nine domains, resulting in a trajectory. The same was done from the CoMs of domains used to calculate the dynamic network of the SARS-CoV-2 spike. We then performed PCA on these trajectories and compared it to the one from the SARS-CoV-2 spike ensemble (Cα atoms), revealing a highly similar profile (Figure S7). Because the PC vectors obtained from each analysis differ in dimensionality, direct calculation of their overlap was not feasible.

However, we assessed the similarity of their respective 2D projections onto the first two PCs using three complementary analyses: *i.* Procrustes, which yielded a disparity score of 0.0049 for the vector model and 0.0151 for the network model (with lower values indicating better alignment); *ii.* Pearson correlation, treating the 2D coordinates as 1D vectors, resulting in a correlation coefficient of 0.998 and 0.994 for the models, respectively; and *iii.* the RMSD, which quantified the average Euclidean distance between corresponding 2D points from the two projections, giving a value of 0.383 and 1.893 Å. Altogether, these analyses show that both CVs (vector-based and derived from the dynamical network) are robust to describe the RBD conformational change. The vector-base pattern is the most similar compared to the PCA from the Cartesian coordinates of the ensemble structures; however, the dynamical network CV has all of the spike residues represented.

To further characterize the conformational landscape of the SARS-CoV-2 spike protein ensemble, an RMSD-based clustering analysis was performed to identify recurrent and distinct structural states. This method effectively grouped similar conformations, enabling the identification of both dominant populations and rare structural variants. The analysis yielded 240 distinct clusters, with the 10 most populated clusters accounting for approximately 80% of all conformations in the ensemble (Figure S8 and Movie S6). The largest cluster alone contained over 1100 structures, representing roughly 40% of the total ensemble. Beyond these prevalent conformations, the analysis revealed around 35 moderately populated clusters and 156 singleton clusters composed of unique structures, highlighting the extensive conformational heterogeneity captured in the ensemble.

### 3.3 Normal Modes Describe Spike Intrinsic Spike Motions

The first two lowest-frequency normal modes calculated from the ensemble reference structure effectively capture the RBD and NTD motions observed in the experimental ensemble (Figure 2A and Movies S2–S5). Notably, when comparing their directions, there is a close correspondence between the normal modes and the first two principal components (PCs): normal mode 2 resembles PC1 (overlap of 0.73), while normal mode 1 corresponds to PC2 (0.68) (Figure 2C). This similarity is also confirmed by comparing individually or collectively the DCCMs derived from the two first PCs and normal modes (Figure S5A). However, this correspondence is less evident when inspecting their RMSF profiles, due to differences in amplitude (Figure 2B), as PC1 alone accounts for approximately 85% of the total ensemble variance (Figure S5B).

The correspondence between intrinsic dynamics and experimental motions of the spike protein was assessed by comparing the first five lowest-frequency normal modes with the essential subspace defined by PCA of the experimental ensemble (Figure S5C). The overlap matrix showed that PC1 aligns best with mode 2 and PC2 aligns best with mode 1, indicating that distinct normal modes capture the principal experimental motions. Projection of both sets onto a common conformational space (Figure 2C) further revealed strong overlap and consistent structural directionality. These findings confirm that low-frequency normal modes effectively reproduce the dominant experimental transitions and provide mechanistic insight into the large-scale conformational shifts of the spike protein.

Normal mode analysis was used to perform domain analysis and predict hinge points in the spike with the *DynDom* server. For modes 1 and 2, three dynamic domains were identified:^1^ residues 29–318 (NTD) and 592–700 (C2);^2^ residues 319–591 (RBD + C1);^3^ residues 701–1145 (S2) (Movies S7 and S8). Predicted hinge points were found at residues 317–323 (RBD), 584–594 (C1/C2), and 706–708 (S2). A similar analysis of two spike experimental structures, 6VXX_A (closed) and 6VYB_B (open), revealed two dynamic domains: one for the RBD and the other for the rest of the protein (Movie S9). The hinge regions align with those observed in cryo-EM maps of spike structures with different RBD conformations.^69^

### 3.4 Ensemble Normal Modes and Signature Analysis of the SARS-CoV-2 Spike

Since proteins exist as dynamic ensembles, ensemble normal modes incorporate this intrinsic plasticity, ensuring that the normal modes align with experimentally observed transitions. In contrast to single-structure NMA, which is highly sensitive to the chosen conformation, ensemble-based methods reduce bias by averaging structural variations.^70–73^

From the ensemble normal modes, signature profiles can be obtained by calculating the mean and standard deviation of properties such as mode shapes and mean square fluctuations. For the SARS-CoV-2, we calculate the average and standard deviation of the shape of the first three modes (individually or collectively) over all conformations (Figure S9). The signature profile of the lowest-frequency mode primarily describes collective motion of the S2 subunit relative to S1. The signature profile of mode 2 captures an NTD/RBD motion, while mode 3 describes a more complex motion of the S2 subunit. The cumulative signature profile and DCCM derived from the first three ensemble normal modes show an anticorrelated motion between S2 and S1; in addition, the RBD and NTD motions are strongly positively correlated.

The main difference between PCs and normal modes (including the ensemble normal modes) lies in the behavior of the S2 domain. Within the experimental ensemble, the S2 domain of one protomer engages in extensive trimeric interactions, forming a large contact area that stabilizes this region. Structurally, the HR1 and CH regions form a continuous α-helix, with their three copies assembling into a long central three-stranded coiled-coil. This tight trimeric interaction (observed in experimental structures) restricts S2 flexibility.^74^ In contrast, in normal-mode analysis based on the protomeric structure, the S2 subunit has fewer contacts and may fluctuate more freely. As a result, normal modes predict greater S2 flexibility compared with PCs, which reflect the constrained motions observed in the experimental ensemble.

To compare modes within the ensemble normal mode data set, spectral overlap, also known as covariance overlap, was used as a more sophisticated measure instead of simple overlap analysis. Additionally, advanced ensemble analysis was performed using matrices based on pairwise metrics: sequence identity (or distance), RMSD, and normal mode similarity (dissimilarity). These matrices were computed for the SARS-CoV-2 ensemble and sorted by spectral overlap distance, RMSD, and sequence identity (see Section 2) (Figure S10). This approach provides a comprehensive assessment of the structural and dynamical relationships among ensemble members.

The RMSD-ordered matrix from the ensemble distinctly separates it into two groups: a major homogeneous cluster of RBD-closed structures and another containing open conformations. When sorted by spectral overlap distance, a smaller subset of structures exhibits greater spectral similarity, while a larger, more populated cluster shows greater spectral variation. Sequence-based comparison further reveals a distinct subgroup with significant sequence divergence—specifically, the Omicron variant, which displays around 40 residue differences from other ensemble members. Altogether, these matrices provide valuable insights into sequence, structural, and dynamic relationships within the SARS-CoV-2 ensemble.

#### 3.4.1 Ensemble Normal Mode-Driven Dynamical Networks Analysis

Normal mode analysis predicts the intrinsic motions a protein can undergo based on its structure. These predicted movements can be used to construct a DCCM, which captures how the motions of different protein regions are related. Regions that tend to move in the same direction exhibit strong positive correlations, while those moving in opposite directions show strong negative correlations. Applying PCA to the DCCM does not yield direct information about physical displacements but instead reveals dominant patterns of dynamic coupling within the structure. In this context, the resulting PCs reflect groups of residues or domains exhibiting coordinated or opposing motion tendencies, as inferred from the ensemble of normal modes. Each PC thus highlights dynamically coupled regions that are potentially involved in concerted functional motions. However, unlike PCA performed on a covariance matrix of atomic displacements, this approach does not provide explicit information about displacement magnitudes or directions.

To investigate the dynamic segregation of functional regions, the values of the first PCs were projected using the DCCM derived from the ensemble of normal modes (Figure S11). PC1 in this space recapitulates the pattern observed in the PC1 derived from the full ensemble analysis of Cartesian coordinates (Figure 1), where PC1 effectively separates the protomers based on the conformational state of the RBD. This agreement indicates that the normal modes capture key differences between structural states, particularly in the RBD, due to distinct dynamical signatures.

To further explore how dynamic coupling among spike residues varies across conformational states, ligand-binding classes, and D614G mutation status, comparative dynamical networks were constructed based on normalized correlation variability, using the DCCM from the normal mode ensemble (Figure 3). In these networks, edge colors indicate residue pairs whose correlations differ significantly across structural groups, revealing an internal dynamic plasticity. In the RBD-closed state (Figure 3A), a dense pattern of highly correlated connections is observed between the RBD and the central helical (CH) domain, located near the trimer’s CoM.

**Figure 3:**
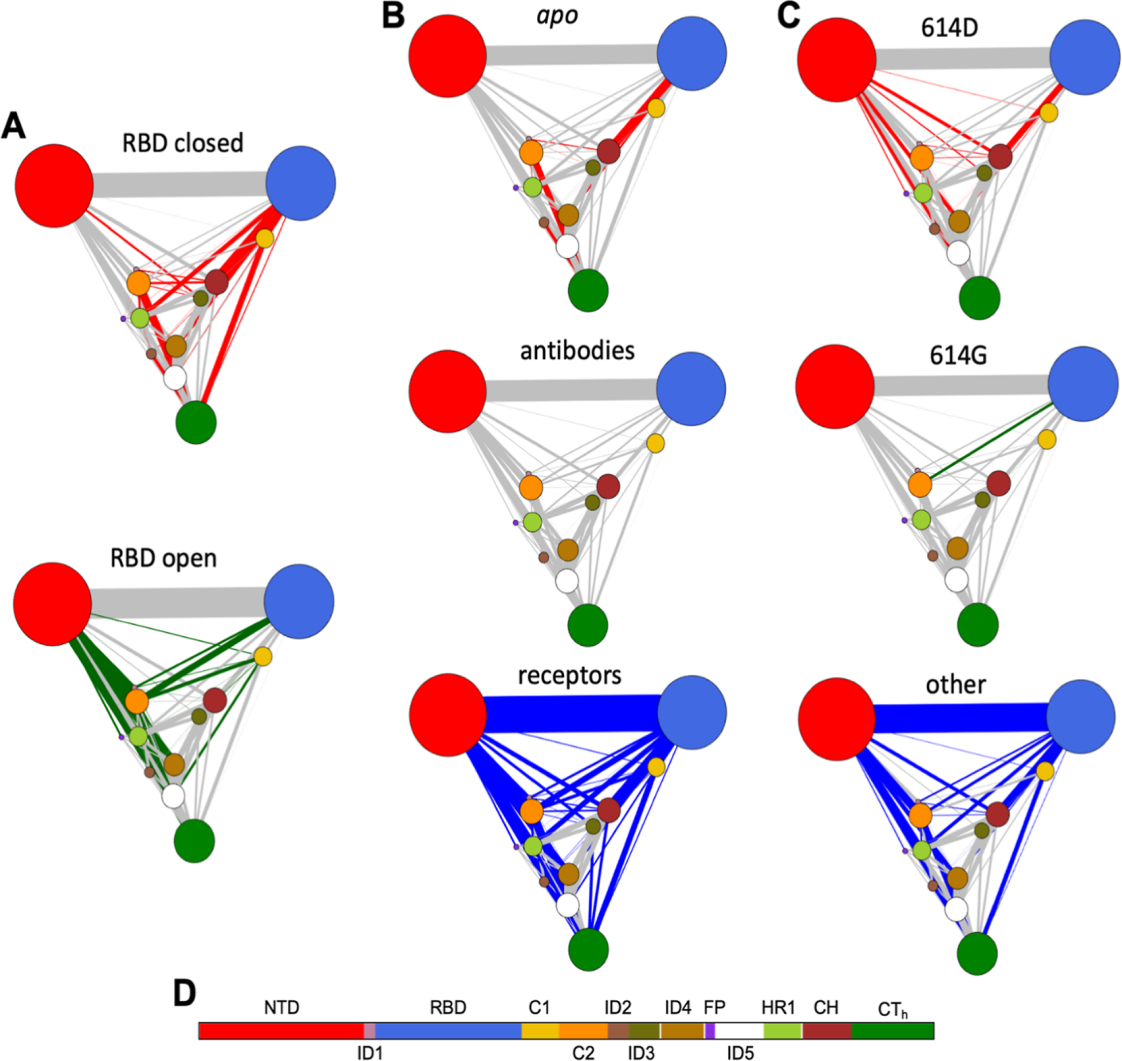
Protein network analysis of the SARS-CoV-2 experimental ensemble. (A–C) Network representations of the SARS-CoV-2 spike protomers from the experimental ensemble: (A) with RBDs in closed and open conformations. (B) Filtered by ligand-binding interactions (e.g., apo, receptors, antibodies); (C) filtered by residue 614 variants (e.g., aspartic acid, glycine, and others). All networks highlight correlated edges, colored by group. (D) A domain bar below annotates the spike regions represented in the networks. Nodes were defined by spike protein domains and interdomain regions: residues 27–303, NTD; 304–318, ID1; 319–541, RBD; 542–591, C1; 592–686, C2; 687–716, ID2; 717–757, ID3; 758–815, ID4; 816–855, FP; 856–919, ID5; 920–970, HR1; 971–1035, CH; and 1036–1147, CT_h_.

This suggests a stabilizing role for the CH domain in the closed conformation. In contrast, the RBD-open network displays a loss of these RBD-CH correlations, with the RBD instead showing stronger connections to other S1 subunit domains. Notably, the NTD exhibits an increased correlation with multiple regions of the protomer in the open state, indicating that the NTD may play a more central role in interdomain communication when the RBD is exposed.

On the other hand, the networks constructed for ligand-bound groups (Figure 3B) clearly show distinct dynamical profiles. Structures bound to receptors, primarily ACE2, exhibit widespread, highly correlated connections across nearly all domains and interdomains, consistent with the substantial conformational rearrangements required to achieve and maintain the open state of the RDB. In contrast, antibody-bound structures show no high correlated connections, reflecting the diversity of antibody epitopes present in the protomer of spike, which can target different spike regions independently of the RBD conformation. *Apo* structures show a more selective pattern with strong correlations limited to a few domains: RBD, C2, and some interdomains, indicating a less flexible trimer state in the absence of ligands.

Lastly, for the groups classified by residue 614 identity (Figure 3C), showing how the D614G mutation and other less seen substitutions or engineered influences spike dynamics. Structures of the other group exhibit a connectivity pattern similar to that of receptor-bound spikes, likely because many of these structures were designed to stabilize particular RBD conformations. The 614D group shows strong connections in NTD and CH domains with some other domains, reminiscent of the tightly packed closed-state network, supporting the idea that this ancestral form favors reduced conformational flexibility. In contrast, the 614G network displays only a single prominent edge colored between the RBD and C2 domains. This marked reduction in dynamic interdomain connectivity may reflect a more flexible or destabilized core that facilitates spontaneous RBD opening, consistent with the enhanced receptor accessibility and infectivity associated with the D614G mutation.

### 3.5 Ligand Binding Modulates Spike RBD Conformation

To evaluate the influence of ligand binding on the spike structure, the ensemble was divided into four categories based on ligand interactions: *apo* (unbound), bound to antibodies, bound to ACE2, and bound to non-ACE2 receptors. Since the Cov3D database provides ligand-binding information at the trimer level, all conformations were reclassified by calculating the number of residues in contact with the ligand. If a conformation has zero contacts, then it is classified as *apo*.

Within the SARS-CoV-2 ensemble, 48.96% of the conformations are in the unbound state (*apo*), while 42.62% are bound to antibodies, 7% are bound to ACE2, and 1.43% are bound to other receptors. Analysis of the projection of each group structure onto PC1, but not PC2, indicates that receptor or ACE2 binding (with a few outliers) stabilizes the spike in an open conformation (Figure 4A,B).

**Figure 4:**
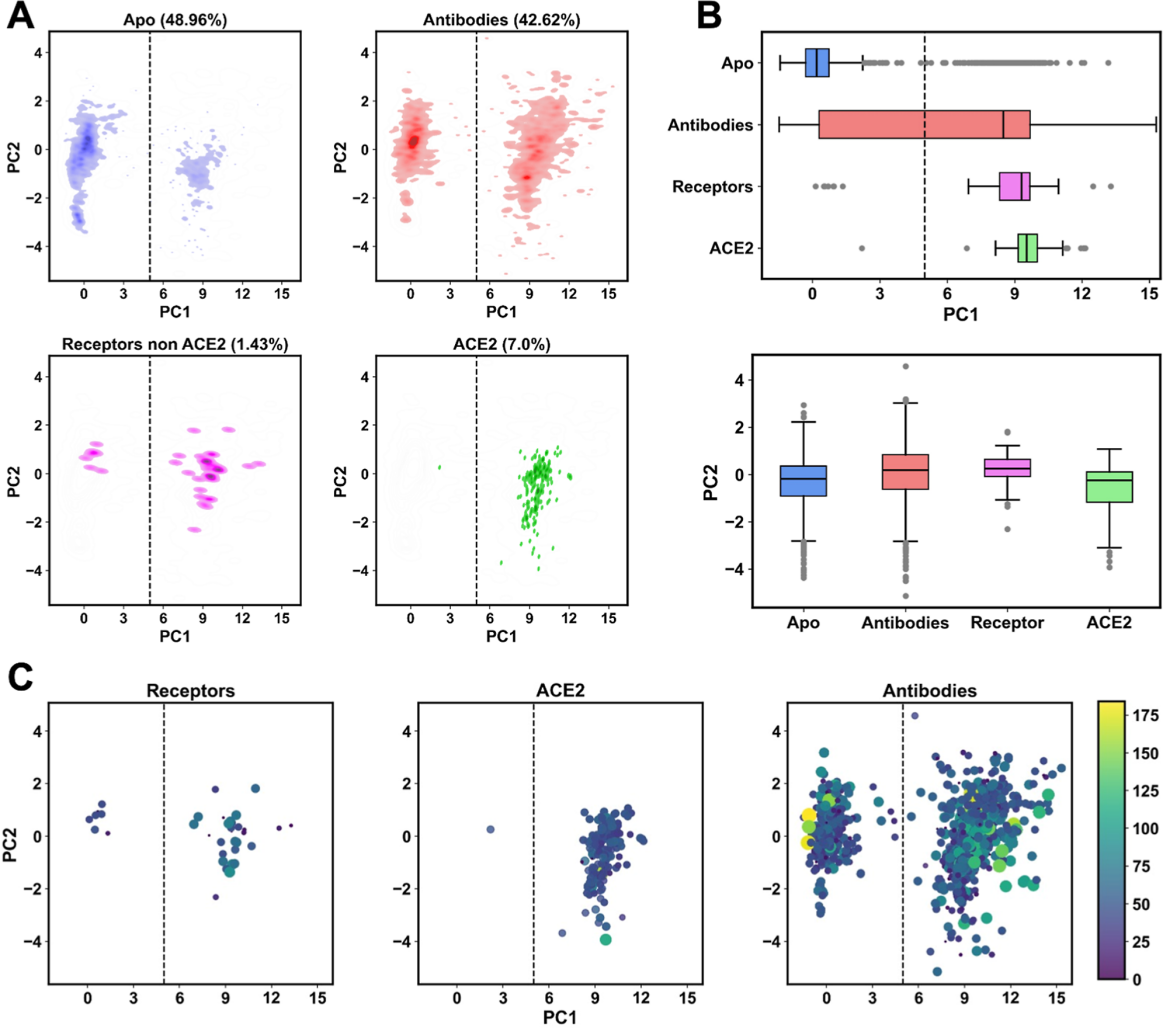
Ligand binding-induced changes in SARS-CoV-2 experimental conformations. (A,B) Distribution of protomeric ensemble structures onto PC1 and PC2 (A) and corresponding boxplots (B): *apo* (blue); and ligand-bound structures, including antibody (red), receptor (pink), and ACE2 (green). Outliers are represented as gray points. (C) Number of spike residues in contact with ligands (within 4 Å) across structures. Marker color (*viridis* scale) and size indicate the number of contacts projected onto the PC 1–2 space.

Among the outliers, only one closed structure bound to ACE-2, which is a mink variant (PDB-ID 8T22), while seven receptor-bound structures (8BON, 7JZN, 7JZL, 7ZRV, 7ZSS, 7KL9, and 7ZSS) also adopt a closed conformation. In contrast, the *apo* conformations predominantly remain in the closed state. Notably, antibodies bind both open and closed RBDs with stronger interactions and more contacts than receptor-bound structures (Figure 4C). Due to their larger size, antibodies can engage multiple RBDs within the trimer. One notable outlier (8W4F) shows high binding (∼180 contacts) with a trivalent nanobody cluster, stabilizing all three chains in the closed state.

All protein–ligand interactions occur within the S1 subunit, primarily in the RBD, which binds the human ACE2 receptor (Figure S12A). Structures complexed with ACE2 interact exclusively within the RBD, focusing on the RBM. In contrast, antibody-bound structures display broader interaction surfaces, engaging an additional domain, the NTD. Analysis of spike interactions with antibody heavy (H) and light chains—*kappa* (K) and *lambda* (L)—reveals a similar binding pattern, predominantly targeting the RBD and interacting with the NTD (Figure S12B). However, the K chain forms fewer contacts with the NTD compared to the H and L chains.

### 3.6 Structural Impact of Spike Mutations

Given the significance of the D614G mutation, which early in the pandemic shifted the spike protein toward a more open RBD conformation,^75,76^ we analyzed RBD conformations in both protomeric and trimeric spike structures under two conditions: *i.* the unbound (*Apo*) form and *ii.* complexed with ligands (*Lig*). Protomer-level analyses are used to isolate the intrinsic effect of ligand binding on individual spike monomers, independent of intersubunit interactions. This approach enables the comparison of RBD conformational preferences across thousands of structures with or without ligand contact. In contrast, trimer-level analyses allow us to evaluate cooperative effects—for instance, how ligand binding to one protomer may influence the conformational state of adjacent RBDs—thus capturing interprotomer coupling that underlies spike functional regulation.

#### 3.6.1 Protomeric Level Analyses

Among the ensemble structures, 45.19% contain the original 614D, while 46.55% carry the 614G mutation. The remaining 8.25% consists of other mutations at the position 614, including engineered variants designed to stabilize specific RBD states (Figure 5A). It was expected that a higher percentage of structures in the 614G group would exhibit the open RBD conformation.^77,78^ However, this number is around 39%, which is similar to that of the 614D group (approximately 43%). In both cases, the closed conformation proved to be more stable.

**Figure 5:**
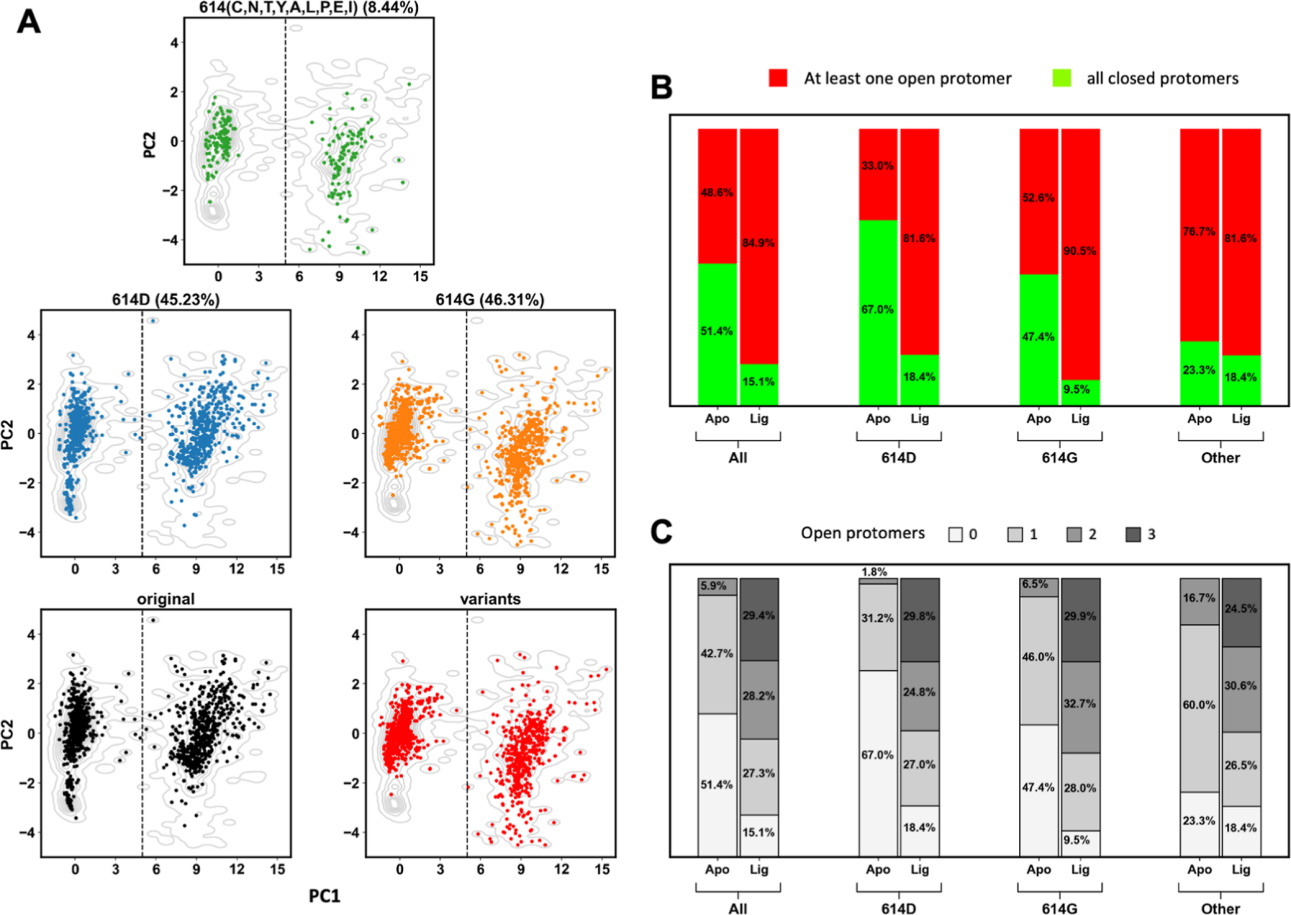
Impact of residue 614 mutation on SARS-CoV-2 spike RBD conformation. (A) Projection of protomeric ensemble structures (classified by the residue at 614 position) onto the PC 1–2 space. Original (Wuhan, black) and variant (red) structures are shown, with gray contour lines representing the ensemble density. (B,C) Distribution of trimeric spike structures by RBD conformation: red (at least one RBD open) and green (all RBDs closed), including apo and ligand-bound forms; (C) grayscale gradients indicate the number of protomers with open RBD conformations.

Although previous studies have reported that the D614G mutation promotes RBD opening and enhances ACE2 accessibility and infectivity, our ensemble-based analysis did not reveal a substantial difference in the frequency of open conformations between the D614 and G614 spike protomers. This apparent discrepancy may be explained in part by methodological differences. First, our analysis is based on experimentally resolved structures deposited in the PDB, which are inherently biased toward ligand- or antibody-bound states—conditions that frequently stabilize open RBD conformations.

As a result, this sampling bias may obscure subtle differences in spontaneous opening propensities between D614 and G614 in the absence of binding partners. Second, our approach evaluates protomeric conformations individually, decoupled from trimeric cooperative effects that may be critical for capturing the functional consequences of D614G substitution. Prior studies reporting increased openness of the G614 spike have often relied on full-trimer cryo-EM classifications or functional assays measuring entry efficiency, which capture aspects of conformational equilibrium and cooperativity not directly reflected in static protomer snapshots. Together, these considerations underscore the importance of interpreting structural ensemble data in the context of their sampling constraints and highlight the value of complementary experimental approaches to validating dynamic and functional hypotheses.

The number of unbound (*apo*) structures is quite different between the two groups: ∼40% 614G; and more than 60% for 614D. From these *apo* structures, around 81% (614D) and 86% (614G) are in the closed conformation, while for the ligand-bound structures, this percentage is around 38% for 614D and 30% for 614G.

To further assess whether the 614G mutation is a defining feature of SARS-CoV-2 variants, we compared the 614D with the original structures and the 614G with the variant strains, revealing a highly similar RBD profile in both cases (Figure 5A), confirming this mutation as a hallmark for SARS-CoV-2 variants.

#### 3.6.2 Trimmer Level

Analysis of all SARS-CoV-2 trimeric spike structures in the ensemble reveals that in the Apo group, 51.4% have all three protomers in the closed RBD conformation, whereas 84.9% of ligand-bound (Lig) structures have at least one RBD in the open state (Figure 5B). These findings highlight the significant impact of ligand binding on the stabilization of the open RBD conformation. Such a ligand effect is similarly observed across spike proteins carrying either the 614D or 614G residue.

For ligand-bound structures, the proportion of fully closed trimers decreases further as expected: only 18.4% of 614D and 9.5% of 614G. These observations support previous studies reporting that the D614G substitution increases RBD accessibility with or without the presence of ligand, likely by destabilizing interprotomer interactions that constrain the closed RBD conformation.^11,76,79^ On the other hand, among *apo* structures, 67.0% of the 614D group remain fully closed, while this proportion drops to 47.4% in 614G, indicating a higher baseline RBD flexibility in the 614G.

Additionally, to investigate other determinants of RBD conformation, we assessed the influence of ligand binding in both the original and mutant structures. To get further insights into how ligand binding and the D614G mutation influence the degree of spike conformational change at the trimer level, we analyzed the distribution of open protomers across groups (Figure 5C). In the *apo* state, the fully open trimers with all three RBDs in the open conformation are absent across all subgroups, suggesting that this configuration is not observed spontaneously. In contrast, ligand-bound structures could adopt the fully open trimeric configuration with certain frequency, with 29.4% of 614D, 29.9% of 614G, and 24.5% of other mutations in this residue. These results confirm that the simultaneous opening of all RBDs is a ligand-stabilized state rather than a spontaneous equilibrium feature, in agreement with cryo-EM studies showing that trivalent engagement (e.g., by three ACE2 or multivalent antibody interactions) promotes and stabilizes this conformation.^80,81^ Moreover, the similar proportions of fully open trimers between 614D and 614G in the ligand-bound group suggest that while the D614G mutation enhances spontaneous RBD opening, it does not significantly alter the extent to which ligands can drive the spike into the fully open state.

Our results reveal that ligand-induced RBD opening does not occur uniformly across the spike trimer but instead follows an asymmetric, locally triggered mechanism. At the protomeric level, RBD opening is strongly associated with the binding of the ligand to that specific subunit. However, at the trimeric level, we observe that full RBD opening involving all three protomers is rare and occurs almost exclusively when all subunits are ligand-bound. In many partially open trimer states (e.g., 1^‑up^ or 2^‑up^ conformations), only the ligand-bound protomer(s) exhibit an open RBD, while unbound protomers remain in the closed conformation. This pattern suggests a model of sequential or asymmetric activation, where ligand engagement facilitates a localized conformational change without requiring full concerted motion across the trimer. Nevertheless, the structural integration of the spike suggests some degree of interprotomer influence, potentially stabilizing or modulating the neighboring subunit behavior. These findings align with allosteric coupling and partial cooperativity in spike activation and might have implications for how spike-mediated receptor engagement and immune recognition are regulated.

### 3.7 Conformational Heterogeneity Across SARS-CoV-2 Variants

The experimental SARS-CoV-2 ensemble was subclassified into variant groups using Cov3D data combined with an MSA-based clustering strategy (see Section 2). The variant-specific structures were selected in the PCs 1–2 space with different colors and the percentage representation of the total ensemble (Figure 6A). Structures from the original (Wuhan) strain have a cluster on negative PC2 values and lower PC1 projections, indicating a closed RBD conformation with minimal interdomain separation between NTD and RBD. This configuration suggests a tighter packing and limited flexibility. In contrast, the Omicron variant populates vastly the same PC2 values but with higher PC1, indicating a new cluster with open RBD conformation but with lower interdomain distance. This observed shift with RBD exposure and lower interdomain flexibility could potentially contribute to altered ACE2 binding kinetics and immune escape strategies.

**Figure 6:**
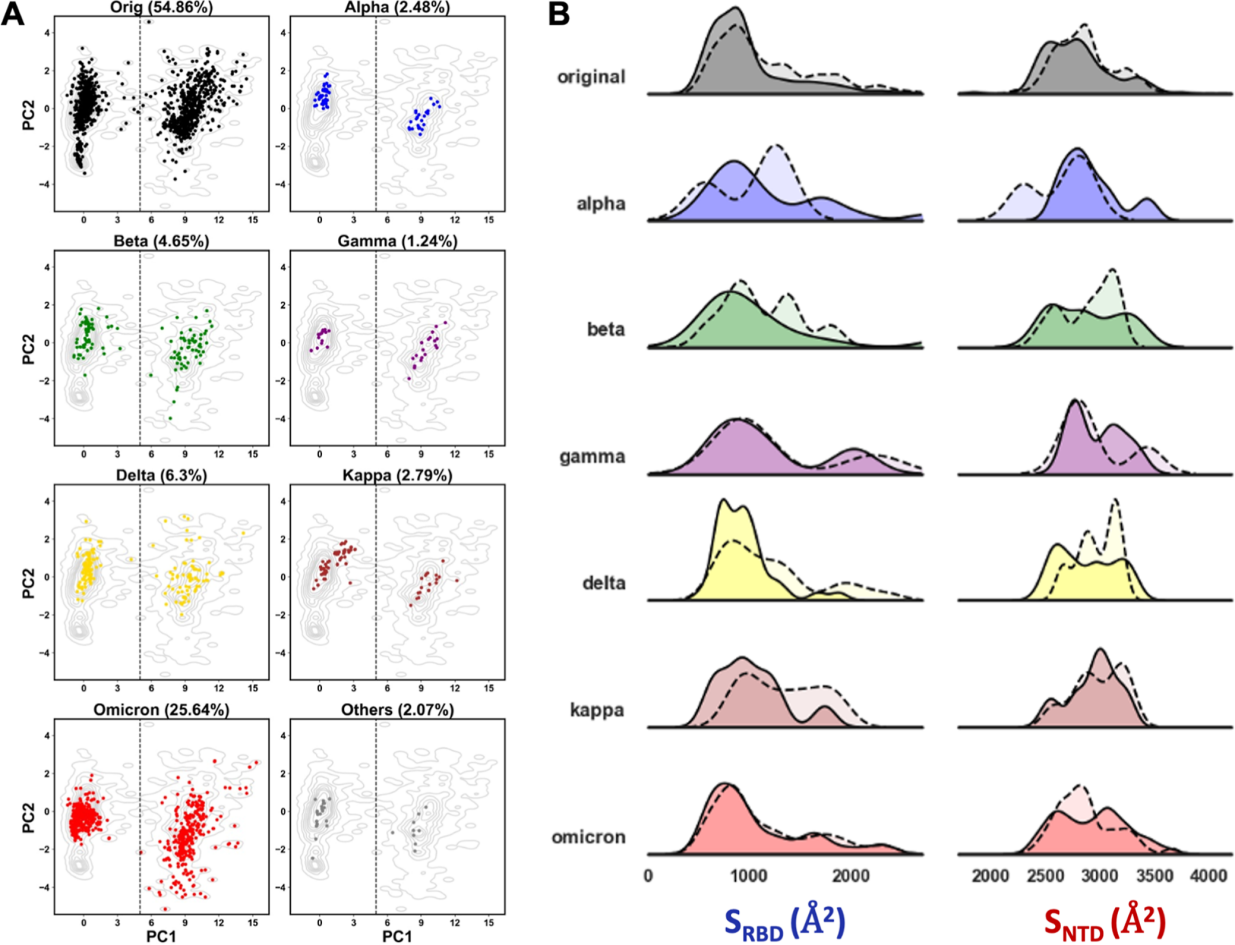
Variant-driven modulation of RBD conformational dynamics. (A) Projection of variant spike structures onto PC1–PC2, colored by variant (percentages are indicated). Gray contours show the ensemble density distribution. (B) Density plots of *S*_RBD_ and *S*_NTD_ areas of trimeric spikes. Solid lines show *apo*, while dotted lines with transparent fill the ligand-bound structures.

To further examine how individual variants distribute within the principal component space, we analyzed the PC1 and PC2 projections across the ensemble using violin plots, separated by RBD conformation (Figure S13). In the PC1 dimension (Figure S13A), structures in the closed RBD conformation generally cluster similarly to the original strain, indicating that most variants maintain a comparable structural baseline in this state.

The Omicron variant shows a modest shift toward more negative PC1 values in the closed state, suggesting a subtle structural compaction. The Kappa variant displays the widest distribution among closed structures and is slightly shifted toward more positive PC1 values, indicating greater conformational variability even without the RBD opening. Among open-state structures, Omicron exhibits the broadest distribution, followed by Gamma and Beta, reflecting increased heterogeneity in their open conformations. Most other variants show PC1 profiles similar to those of the original strain in the open state, with the Alpha variant slightly skewed toward lower values.

In the PC2 dimension (Figure S13B), the original strain presents the widest distribution overall, but its closed and open structures have overlapping profiles centered near zero, suggesting limited interdomain variation between states. For most variants, PC2 distributions differ more distinctly between the closed and open states. A general trend emerges where closed structures tend to have higher PC2 values, while open structures shift toward lower PC2 values. The Omicron variant stands out with the most pronounced separation: its closed-state structures show a narrow distribution at moderately negative PC2 values, while its open-state structures expand into the lowest PC2 space. This indicates a marked conformational reorganization upon RBD opening, reinforcing the notion that Omicron exhibits distinct dynamics compared to the original strain and other variants.

To dissect these conformational differences more precisely, we evaluated collective variables that describe the trimer architecture in both *apo* and ligand-bound states, as previously proposed 50. Specifically, we calculated the areas formed by the CoMs of the RBDs (*S*_RBD_) and NTDs (*S*_NTD_) in each trimeric structure (Figure 6B). The *S*_RBD_ distributions in the *apo* structures revealed one to three distinct populations, depending on the variant. Among them, the original, Delta, and Kappa variants exhibited narrower distributions with lower variance, indicative of more stabilized trimer in the absence of ligands. For most variants, including Alpha, Beta, and Delta, ligand binding induced a broader distribution with additional peaks and a dislocation to higher areas, suggesting enhanced flexibility and RBD rearrangements in response to binding events. However, for the original, Omicron, and Gamma variants, the *S*_RBD_ distributions remained largely unchanged upon ligand binding, implying reduced conformational plasticity or a pre-existing structural equilibrium that favors specific RBD orientations.

In the case of *S*_NTD_, the original variant again showed minimal differences between the *apo* and ligand-bound states, reinforcing the notion of a structurally rigid NTD arrangement. For most variants, ligand binding led to a moderate increase in the *S*_NTD_ values, consistent with NTD expansion often associated with trimer opening. Interestingly, the Omicron and Alpha variants deviated from this trend: their *S*_NTD_ distributions shifted toward lower values upon ligand binding, suggesting a more compact NTD configuration. This observation points to a variant-specific structural adaptation in which ligand interaction may induce local compaction in some spike domains rather than global expansion. Together, these results highlight distinct conformational behaviors across variants, with Omicron in particular showing limited change in both *S*_RBD_ and *S*_NTD_ upon ligand binding, with a possible preorganized conformation that supports efficient receptor engagement and immune evasion.

#### 3.7.1 Single-Experiment Cryo-EM Multiple Metastates of the Beta Spike Variant

Recent advances in cryo-electron microscopy (cryo-EM) have made it possible to resolve multiple conformational states (metastates) of macromolecular complexes within a single experimental setup, capturing both discrete and continuous structural variations.^82–86^ Among the SARS-CoV-2 spike glycoprotein structures deposited in the PDB, two entries—9GDX and 9GDY—stand out for containing multiple models. These models correspond to the spike protein of the Beta variant (B.1.351), derived from samples equilibrated during vitrification at two distinct temperatures: 4 °C (9GDX) and 37 °C (9GDY). These multiple models were generated using a deep learning-based framework, HetSIREN, designed to resolve structural heterogeneity by learning a low-dimensional conformational latent space directly from particle images.^86^

In our analysis of the SARS-CoV-2 spike ensemble, we automatically considered only the first model of each entry. However, to fully explore the conformational landscape captured by the cryo-EM-derived multiple models, we constructed a new ensemble comprising all 20 models from each PDB entry (9GDY and 9GDX), resulting in a total of 120 conformations (considering all protomers). We compare this ensemble with the entire SARS-CoV-2 ensemble and with its beta variant conformations (43 trimmers, around 130 conformations). First, we performed a PCA over the cryo-EM multimodel ensemble, and the first two PCs presented a high overlap with those from the entire SARS-CoV-2 ensemble, 0.98 and 0.86, respectively (Figure S5D). The PC projection of each structure on ensemble subspace revealed a similar profile of the 9GDX models when compared to the beta variant from the SARS-CoV-2 ensemble (Figure S14A), presenting a mix of closed and open structures.

The 9GDX ensemble revealed a mixture of 1^‑up^ and 2^‑up^ conformations of the spike trimer, whereas in 9GDY, a distinct conformational landscape dominated by the 3_‑down_ state was observed. Moreover, 9GDY presented a minor population adopting the 1^‑up^ conformation (Figure S14B). Beyond the differences in RBD conformations, additional temperature-dependent structural variability was observed in the NTD, which was particularly pronounced in the 9GDY structures. This ensemble sampled structures with more negative values of PC2, which correspond to NTD/RBD motions (Figure S14C). Notably, the 1^‑up^ conformation at 37 °C exhibited a more restricted opening compared to its counterpart at 4 °C, underscoring the influence of temperature on the conformational flexibility and metastability of the spike trimer.

#### 3.7.2 Structural and Functional Variability of Omicron Subvariants

To investigate conformational variability within the Omicron lineage, we projected the subvariants onto the same PV space (PC1–PC2) used for the global ensemble analysis (Figure S15). In the low-PC1 region associated with the closed RBD conformation, all Omicron subvariant structures remain confined to a similar zone, indicating structural conservation in this state. In contrast, in the high-PC1 region associated with the open RBD conformation, the sublineages diverge more visibly. BA.1 and BA.2 subvariants extend into the extreme negative range of PC2 values, while XBB subvariants occupy a region with the highest PC1 values among the Omicron group. These patterns suggest that although all Omicron subvariants share a preference for RBD openness, they exhibit subtle conformational differences that may reflect mutations affecting the dynamics of the protomer. Notably, the early sublineages appear to contribute more strongly to the exploration of extreme PC2 values, a feature that is diminished or absent in more recent sublineages such as XBB, pointing to a potential evolutionary narrowing of the conformational landscape within the lineage.

#### 3.7.3 Omicron Variant Displays Distinct Dynamics

The Omicron variant of SARS-CoV-2 present in the experimental ensemble displays markedly dynamic behavior compared to other variants, as revealed by ensemble NM-based dynamical network analyses (Figure 7A). The generated dynamical networks for both Omicron and non-Omicron spike structures with the same communities revealed substantial differences within the S1 subunit.

**Figure 7:**
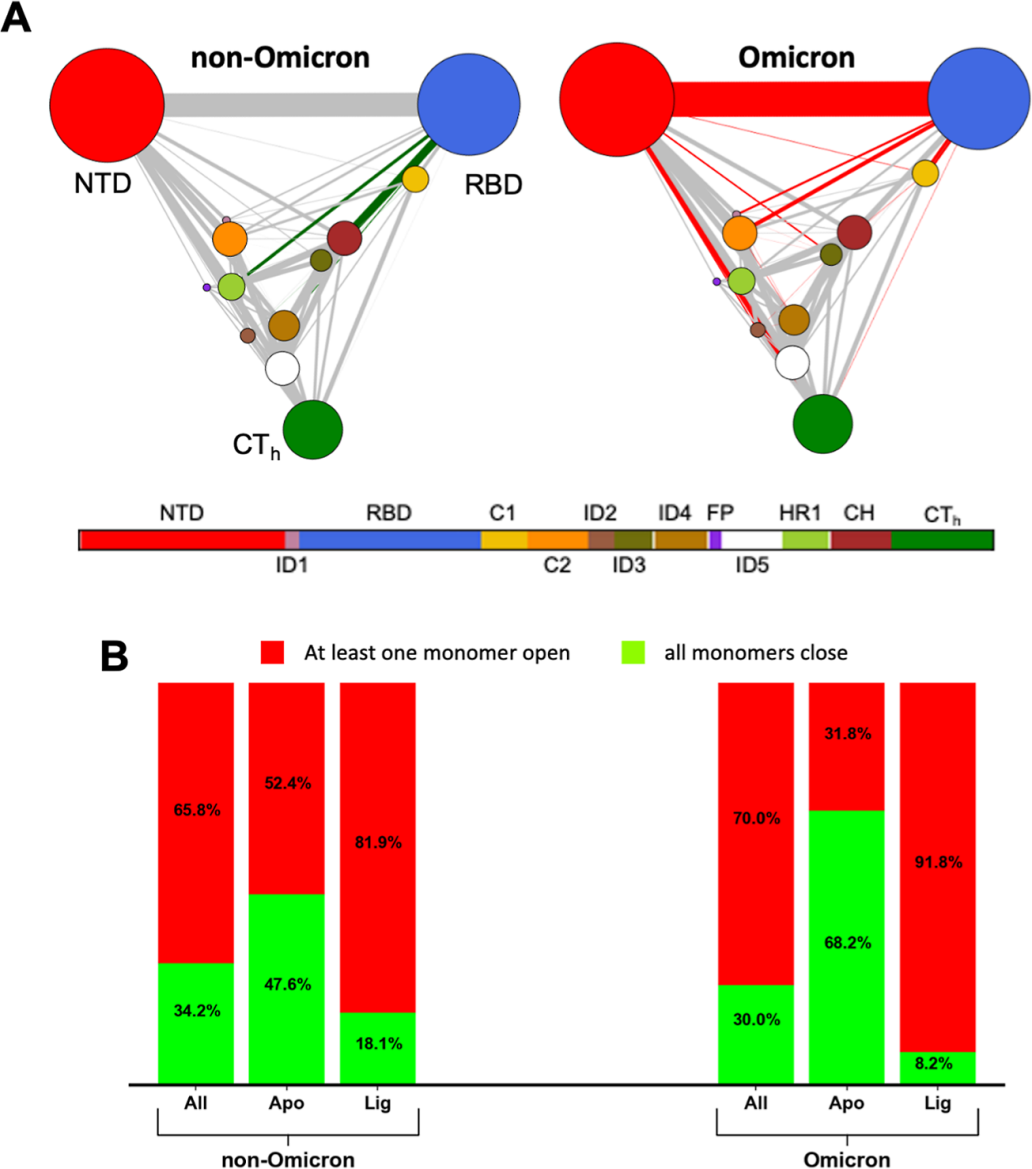
Omicron variants display distinct RBD dynamics. (A) Dynamical network analysis of Omicron and non-Omicron variant structures, highlighting differences in interdomain correlations. Communities were remodeled according to the dynamical domain bar shown below the plots. Nodes were defined by spike protein domains and interdomain regions: residues 27–303, NTD; 304–318, ID1; 319–541, RBD; 542–591, C1; 592–686, C2; 687–716, ID2; 717–757, ID3; 758–815, ID4; 816–855, FP; 856–919, ID5; 920–970, HR1; 971–1035, CH; and 1036–1147, CT_h_. (B) Comparison of RBD conformations of trimeric structures (all, *apo*, and ligand-bound): red (at least one RBD open) and green (all RBDs closed).

In Omicron structures, interdomain correlations between NTD, RBD, and neighboring subdomains were notably altered, getting more correlated, and paths that communicate between RBD and the S2 subunit were more weakened, suggesting a rearrangement of collective motions. These changes likely arise from the high number of mutations in Omicron, compared to other variants and the Wuhan spike. Notably, mutations such as S371L, S373P, and S375F have been implicated in enhancing the stability of RBD conformations, thereby modulating the spike flexibility.^87^ These alterations may contribute to Omicron’s ability of the omicron to evade neutralizing antibodies while maintaining efficient ACE2 binding.

##### 3.7.3.1 *Apo* Omicron Variant Preferentially Stabilizes RBD in the Closed Conformation

Complementing the network analysis, a bar plot comparison of RBD conformational states within trimeric spike structures of the structures of the SARS-CoV-2 ensemble is shown in Figure 7B. These data indicate that the Omicron variant predominantly adopts a closed RBD conformation in its *apo* form, contrasting with other non-Omicron structures that more frequently exhibit open RBD states. This closed state preference in Omicron is consistent with the cryo-EM study showing a higher proportion of spike proteins with all RBDs in the down position, which may facilitate immune evasion by concealing key epitopes.^88^ However, upon binding to ligands, Omicron’s spike protein can transition to open RBD conformations more easily than the other group, indicating that ligand engagement can overcome the conformational constraints imposed by its mutations, consistent with ref 89. These findings underscore the dynamic adaptability of the Omicron spike protein, balancing immune evasion with receptor accessibility.

##### 3.7.3.2 Omicron Interactions with RBD (or Non-RBD)-Targeting Antibodies

To assess how Omicron’s conformational plasticity may influence epitope accessibility and immune recognition, we performed a structural analysis of spike–antibody complexes using the Cov3D classification system and epitope definitions from Chen et al.^90^ and Barnes et al.^91^ Antibodies were grouped into RBD-targeting classes (Classes 1–4, with Classes 1 and 2 specifically engaging the RBM) and non-RBD-targeting categories (NTD, SD1, NTD/SD2, and S2). We compared the Omicron and non-Omicron variant complexes to identify potential differences in conformational preferences and contact patterns.

As shown in Figure S16A, Class 1 and 4 antibodies preferentially bind to open RBD conformations, whereas Class 2 and 3 antibodies engage in both open and closed states. Antibodies targeting the S2 region primarily interact with glycans in the HR1 domain and exhibit minimal direct contact with the spike protein surface. Despite Omicron’s distinct conformational preference for the RBD-closed state in the apo form, we did not observe statistically significant differences in the number of spike–antibody contacts between Omicron and non-Omicron variants across epitope classes (Figure S16B). A residue-level comparison of spike–antibody interfaces (Figure S17) revealed largely conserved contact patterns, with the most notable difference observed in NTD-targeting antibodies: Omicron structures exhibited increased interaction with residues located at the N-terminal region of the domain (with a peak around residue 82).

Overall, these findings indicate that the Omicron retains the established class-specific binding modes of antibody engagement, while modest shifts in NTD epitope usage may contribute to immune evasion. Importantly, prior studies have demonstrated that targeting the RBM specifically mimicking the ACE2-binding interface can be an effective antiviral strategy.^92^ Our results highlight that conformational accessibility of RBM-targeted epitopes depends on dynamic RBD motions and variant-specific stabilization patterns, which should be considered in therapeutic design.

The dynamic features identified in this study have potential applications for the rational design of vaccines and therapeutics targeting SARS-CoV-2 and emerging variants. Stabilization of the spike protein in a specific conformational state, particularly the RBD-closed form, has been a key strategy in current vaccine development. Our findings reinforce that the Omicron variant naturally favors this closed conformation, suggesting that vaccine immunogen design must account for altered epitope exposure and reduced interdomain flexibility. Conversely, therapeutic antibodies may need to target conserved or cryptic epitopes accessible in both closed and transiently open states or exploit the identified hinge regions and interdomain correlations to destabilize functionally essential conformations.

### 3.8 Characterizing the RBD Transition Landscape through MD Simulations

#### 3.8.1 Standard Microsecond-Long MD Simulations Fail to Sample RBD Open/Closed Transition

We analyzed two 10 μs-long standard MD simulations of the SARS-CoV-2 trimer spike protein starting from all_‑down_ and 1^‑up^ RBD structure (PDB-IDs 6VXX and 6VYB, respectively), projecting the trajectories onto the essential space defined by the PC1–PC2 space (Figure 8A). In both cases, the conformational sampling remained confined near their respective initial states, with no significant transitions observed between closed and open RBD conformations. Simulation 1 (starting from 6VXX) explored a narrow region corresponding to closed conformations, while simulation 2 (starting from 6VYB) had chain B (RBDup) remain trapped within an open region without the transition to a closed state. These results highlight the inherent limitations of conventional MD simulations in capturing large-scale conformational transitions of the spike protein within accessible time scales, emphasizing the need for enhanced sampling strategies to explore its functional dynamics more completely.

**Figure 8:**
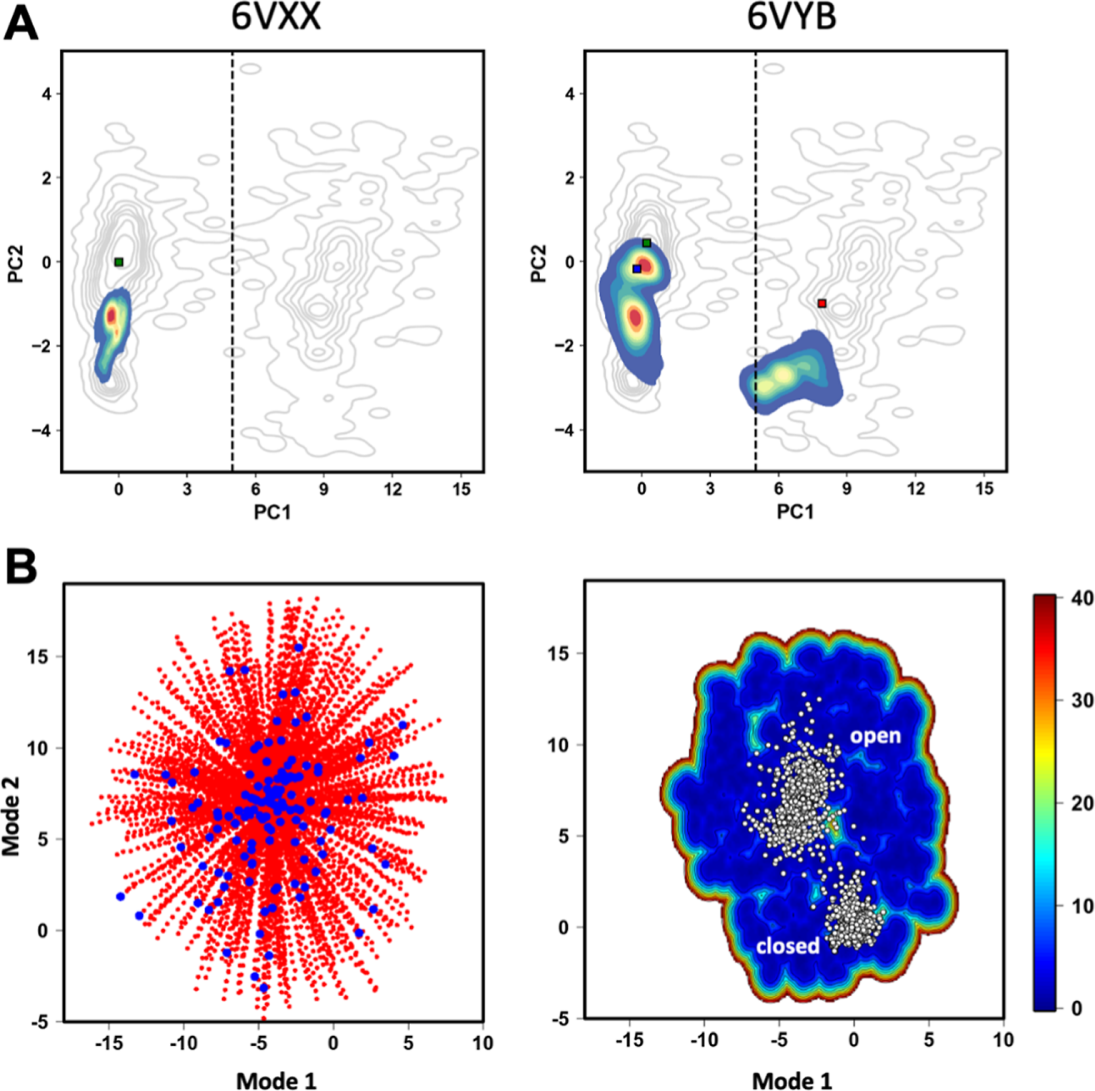
Conformational sampling of RBD transitions by standard and hybrid MD. (A) PCA projections of two 10 μs standard MD simulations starting from different structures (6VXX: left; 6VYB: right), shown as jet-colored density contours. Gray contours represent experimental ensemble projections. (B) Hybrid NM-based MD trajectories (red dots) projected onto the first two normal modes, with representative clusters shown as blue dots. Right: corresponding FEL illustrating the thermodynamic distribution of sampled conformations. Light gray dots indicate experimental ensemble structures.

#### 3.8.2 Hybrid MD Simulations Fully Explore Spike Experimental Conformational Space

A recent benchmarking study^93^ highlighted MDeNM’s capacity to reproduce large-scale conformational changes in complex biomolecular systems, reinforcing its value as a predictive tool for studying protein flexibility and function. Notably, metadynamics simulations using normal modes as CVs show good agreement with MDeNM results, although MDeNM tends to be more computationally efficient.^58^ Furthermore, MDeNM has been demonstrated to effectively capture protein dynamics on the microsecond time scale,^59^ making it a powerful approach for sampling slow conformational transitions. A comprehensive overview of NM-based enhanced sampling methods is provided in ref 94.

To overcome the sampling limitations observed in standard MD simulations, we employed MD with excited normal modes (MDeNM)^58^ to explore the conformational landscape of the SARS-CoV-2 spike protein (Figure 8B). MDeNM trajectories efficiently sampled a broad range of conformations, encompassing both closed and open RBD states observed experimentally. Clustering of these trajectories revealed representative structures that span the full extent of the essential space defined by the first two normal modes. Furthermore, an FEL constructed from the simulations of representative structures demonstrated all of the space present in the experimental SARS-CoV-2 ensemble.

In summary, we highlight that standard MD simulations initiated from either the all-closed or partially open (1^‑up^) conformations remain kinetically trapped around their starting states and fail to explore transitions between open and closed RBD conformations, even over 10 μs time scales. In contrast, MDeNM, by selectively injecting energy along low-frequency normal modes, efficiently samples both closed and open conformations, fully covering the conformational space observed in experimental structures. This underscores the predictive and exploratory power of MDeNM in capturing large-scale, low-probability transitions that are functionally relevant for spike activation but not accessible by standard MD under practical time scales.

These findings demonstrate that hybrid MD simulations are capable of capturing the full range of experimentally observed spike dynamics, providing a powerful tool for studying the conformational flexibility critical to spike function and viral infectivity. Moreover, the use of enhanced sampling techniques, such as MDeNM, to predict structural flexibility could guide the identification of allosteric sites or conformational traps, expanding the repertoire of antiviral strategies beyond the receptor-binding interface.

## 4 Conclusions

In this study, we conducted a comprehensive structural and dynamical analysis of HCoV spike proteins with a particular focus on SARS-CoV-2 and its variants. Our investigation was driven by the central question of how sequence variation, ligand interactions, and environmental factors shape the conformational plasticity of the spike protein and how these changes could impact viral infectivity, immune escape, and therapeutic design.

By constructing and analyzing a large ensemble of experimentally determined structures (over 3000 conformations), we demonstrated that despite considerable sequence divergence, spike proteins from different HCoVs exhibit a conserved capacity to sample both closed and open RBD conformations. For SARS-CoV-2, PC and normal-mode analyses revealed that the dominant conformational transition involves a hinge-like motion of the RBD relative to the NTD. This movement is captured by PC1 and is strongly correlated with geometric descriptors such as RMSD, interdomain distance, and RBD angle, all of which describe the same essential transition.

Importantly, our analyses showed that ligand binding is consistently associated with open RBD conformations in both protomeric and trimeric spike structures. The strong correlation between ligand engagement and RBD opening supports a model in which binding events contribute to stabilizing functionally relevant open states. Our separation of protomer and trimer analyses allowed us to assess the intrinsic influence of binding on individual protomers while also exploring potential cooperative effects within the trimer, including the impact of ligand binding in one protomer on the conformational states of neighboring subunits.

Nonetheless, our ensemble strategy also presents a series of limitations. PDB structural data are inherently biased toward ligand-bound or antibody-bound spike conformations, which may overrepresent open RBD states and underrepresent functionally relevant, unbound open states. Additionally, structural heterogeneity in experimental conditions and construct design (e.g., stabilizing mutations) could influence the observed conformational distributions. While we attempt to account for these factors through stratified analyses and enhanced simulations, such biases must be considered when generalizing conformational trends across variants or inferring functional mechanisms from structural ensembles alone.

A key outcome of this study is the identification of variant-specific conformational and dynamical signatures. Notably, the Omicron variant adopts a unique compact open-RBD conformation with reduced interdomain separation, a state not previously described. Our ensemble-based network analysis further revealed rewiring of allosteric communication pathways in Omicron, including weakened coupling between the RBD and S2 subunit, alongside increased correlation within S1 domains. In contrast, the broader conformational landscape observed in variants such as Delta and Gamma may reflect a more balanced trade-off between immune exposure and receptor accessibility. These dynamic signatures underscore how SARS-CoV-2 variants fine-tune spike plasticity to optimize fitness. Furthermore, the identification of hinge regions and correlated domain motions across variants could inform vaccine strategies aimed at stabilizing the spike in specific conformations or guide therapeutic design targeting motion-restricting regions to impair viral entry.

Using single-experiment multimodel cryo-EM data sets from the Beta variant, we captured metastable states of the Beta variant spike, with a clear temperature-dependent shift between open and closed RBD conformations. Comparison with the broader SARS-CoV-2 ensemble revealed that the low-temperature cryo-EM multimodels closely matched the distribution and essential dynamical space occupied by Beta variant structures. This strong correspondence validates both ensemble-based and single-experiment multiple-state cryo-EM approaches for accurately capturing spike conformational heterogeneity and metastability, suggesting that targeting metastable states may be a viable strategy.

Our findings also contextualize the effects of the D614G mutation, showing that while this substitution may modestly increase the population of open RBD conformations, ligand binding remains the stronger determinant of the RBD state within both mutant and wild-type structures. Furthermore, we demonstrated that trimeric spike structures with three RBDs in the open state are observed only in the presence of ligands, underscoring the cooperative nature of spike activation.

Finally, we showed that standard MD simulations fail to capture large-scale RBD transitions within accessible time scales, whereas our hybrid simulations using MdeNM successfully sampled the full range of experimentally observed conformations. This highlights the predictive power of our ensemble-based hybrid approach for exploring spike flexibility and identifying potential conformational intermediates of functional relevance. Altogether, this work provides mechanistic and structural insights into the conformational behavior of SARS-CoV-2 spike variants, revealing how mutations, binding events, and environmental factors dynamically shape spike function. These findings contribute to our understanding of variant-specific viral adaptation and offer valuable directions for the rational design of vaccines and therapeutics that target the conserved and flexible regions of spike protein.

## Supplementary Material







## Data Availability

The scripts used to download, process, and build the structural ensemble of SARS-CoV-2 spike glycoprotein structures—comprising 72 conformations derived from the 20 most representative PDB cluster entries—are available at: https://github.com/yago52/tutorial_spike. This repository also includes the full ensemble of SARS-CoV-2 spike structures, as well as scripts for analysis, plotting, and dynamical network analysis. Additionally, the structures, configuration files, and input scripts required to perform the MDeNM simulations are provided and can be accessed directly at: https://github.com/yago52/tutorial_spike/blob/main/mdenm_spike.tar.gz. Two 10 μs molecular dynamics trajectories of the trimeric SARS-CoV-2 spike protein, generated by D. E. Shaw Research, were obtained from the COVID-19 Molecular Structure and Therapeutics Hub (https://covid.molssi.org/org-contributions/) and are available under the data set codes DESRES-ANTON-11021566 and DESRES-ANTON-11021571.

## Notes

Spike structural organization, analysis workflows, PCA and NM comparisons, clustering results, residue contact maps, and conformational analyses by variant (PDF)
Molecular animations visualizing SARS-CoV-2 spike ensemble dynamics, PC motions, NM displacements, representative cluster structures, and domain analysis (ZIP)
Annotation of HCoV spike protein PDB structures used in the study, including resolution, chain ID, organism, RBD state, ligand information, and classification (XLSX)

## §

Faculdade de Tecnologia, Universidade Estadual de Campinas, 13484-332, Limeira, São Paulo, Brazil
