# Supplementary material for: Exploring the Intrinsic Structural Plasticity and Conformational Dynamics of Human Beta Coronavirus Spike Glycoproteins
Source: J Chem Inf Model. 2025 Jul 17;65(14):7712–33. doi: 10.1021/acs.jcim.5c00990 (PMC12308813; doi:10.1021/acs.jcim.5c00990)
Supplement: Supplementary file 1 [file ci5c00990_si_001.pdf]

Supporting Information

Exploring the intrinsic structural plasticity and conformational dynamics of beta coronavirus spike glycoproteins

Yago F. Silva[1,2] 0009-0005-2781-1203, Harold H. Fokoue[1,§] 0000-0001-7823-127X and Paulo R. Batista[1,2,*] 0000-0002-3399-174X

[1] Programa de Computação Científica, Vice-Presidência de Educação, Informação e Comunicação, Fundação Oswaldo Cruz. Av. Brasil 4365, Residência Oficial, Manguinhos. 21040-900, Rio de Janeiro, Brasil.

[2] Programa de Pós-graduação em Biologia Computacional e Sistemas, Instituto Oswaldo Cruz, Fundação Oswaldo Cruz. Av. Brasil 4365, Manguinhos. 21040-900, Rio de Janeiro, Brasil.

[§] Present address: Universidade Estadual de Campinas, Faculdade de Tecnologia, 13484-332, Limeira, São Paulo – Brasil.

*To whom correspondence should be addressed: pbatista@fiocruz.br

GitHub repository description. The scripts to download, build the ensemble for a subset of the SARS-CoV-2 structures (72 conformations from the PDB entries of the 20 most representative clusters), analyze, plot and perform the dynamical network analysis are available in the GitHub (https://github.com/yago52/tutorial_spike). The ensemble of all SARS-CoV-2 structures is also included.
The structures, configuration files and scripts to perform the MDeNM simulations are also provided (https://github.com/yago52/tutorial_spike/blob/main/mdenm_spike.tar.gz).

Supplementary Table

Table S1. Annotation of HCoVs PDB structures used in this study. The table provides detailed information on all structures included in the ensembles of human beta-coronavirus spike proteins. Each page is dedicated to a specific organism, with color coding applied for clarity: SARS-CoV-2 (green), SARS-CoV (yellow), MERS-CoV (blue), HKU1 (orange), and OC43 (violet). The table includes the following data for each structure: PDB-ID, chain-ID, resolution (Å), organism, title, or description of the PDB entry, reference DOI, RBD state (*e.g.*, open, or close), variant of the spike protein, ligand state (*e.g.*, *apo*, ACE2, receptor or antibody), and the ligand(s) name.

Supplementary Movie captions

Movie S1. SARS-CoV-2 ensemble ordered by RMSD illustrates RBD opening. Visualization of the experimental spike ensemble ordered by increasing RMSD. Structures are shown as white tubes, with RBD and NTD highlighted as quick surf representations in blue and red, respectively.

Movie S2. Primary spike RBD motion described by PC1. Visualization of PC1-derived motion from the experimental SARS-CoV-2 spike ensemble, resembling a hinge-like outward rotation of the RBD to expose the ACE2-binding surface. The spike is shown as a white tube (reference: 6VXX, chain A), with RBD and NTD highlighted as blue and red quick surf surfaces, respectively. Displacements are scaled to 5 Å.

Movie S3. NTD/RBD interdomain motion described by PC2. Visualization of the motion described by PC2 scaled by 5 Å, corresponding to RBD-NTD interdomain motion, derived from the experimental ensemble of the SARS-CoV-2 spike protein. Spike representation as in Movie S2.

Movie S4. Normal mode 1 predicts NTD/RBD interdomain motion. Visualization of the first non-trivial normal mode, rescaled by 5 Å, showing NTD–RBD interdomain motion similar to PC2 from the experimental ensemble. Spike representation as in Movie S2.

Movie S5. Normal mode 2 predicts primary RBD motions. Visualization of the second non-trivial normal mode, rescaled by 5 Å, showing RBD hinge-bending motion derived from the experimental SARS-CoV-2 spike ensemble. Spike representation as in Movie S2.

Movie S6. Superposition of the representative ensemble structures of the 10 most-populated clusters. Tube visualization of the representative structures from the 10 largest RMSD-based clusters, aligned by the S2 region and shown as red tubes. The animation includes continuous rotation to highlight structural variations.

Movie S7. Dynamical domain analysis and hinge detection from normal mode 1. Morphing trajectory from the initial to 5Å mode 1-displaced structure, highlighting dynamic domains (blue, red, yellow tubes) and hinge regions (green) identified by *DynDom*. White arrows indicate rotation axes.

Movie S8. Dynamical domain analysis and hinge detection from normal mode 2. Morphing trajectory from the initial to 5Å mode 2-displaced structure, highlighting dynamic domains (blue, red, yellow tubes) and hinge regions (green) identified by *DynDom*. White arrows indicate rotation axes.

Movie S9. Dynamical domain analysis and hinge detection from close and open RBD experimental structures. Morphing trajectory from the close (6VXX_A) to open (6VYB_A) experimental structures, highlighting dynamic domains (blue, red tubes) and hinge regions (green) identified by *DynDom*. White arrows indicate rotation axes.

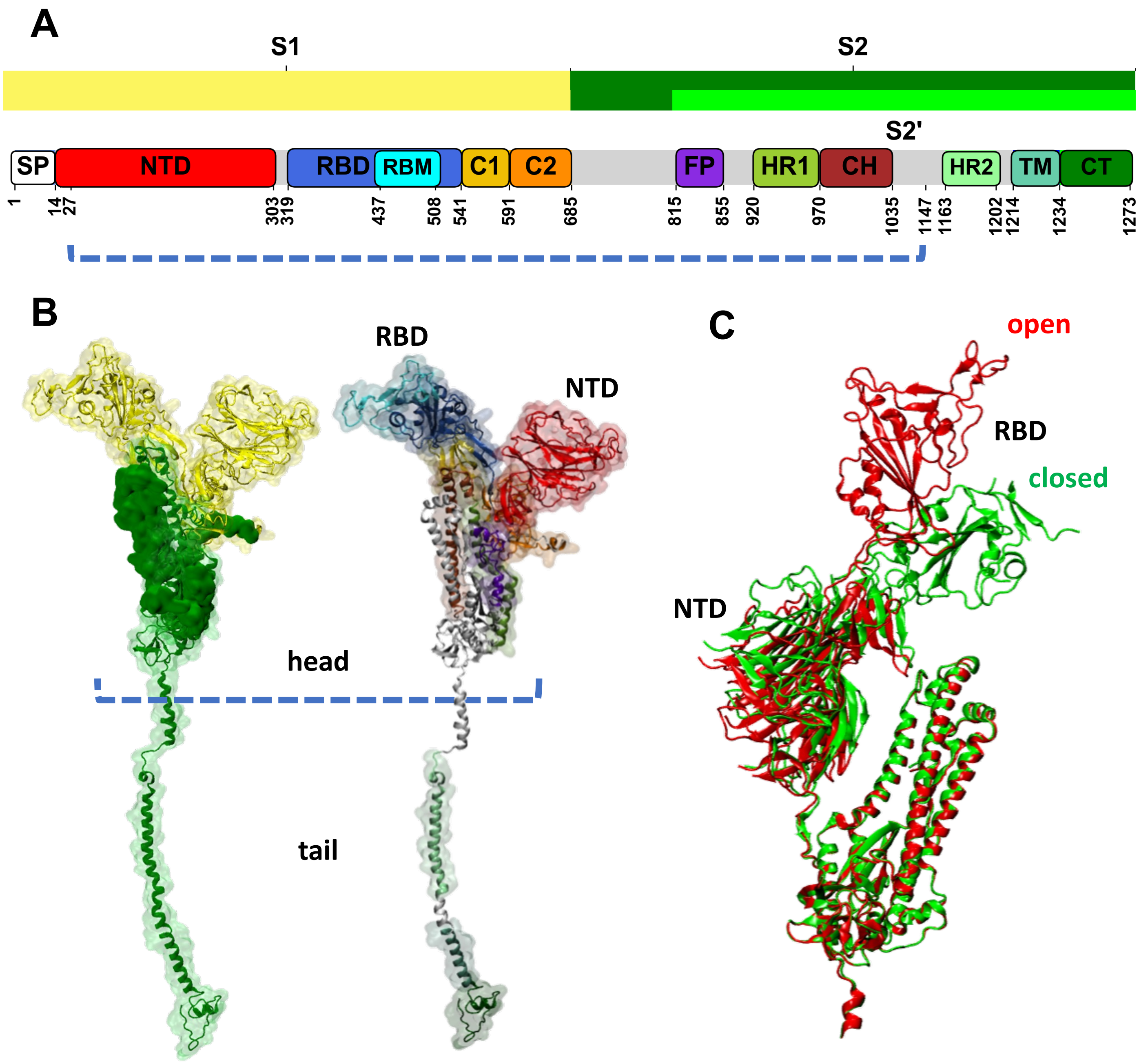

Figure S1. Structural organization of the SARS-CoV-2 spike protein. (A, B) Schematic of the SARS-CoV-2 spike protein domains. (A) Top: subunit organization: S1 (gold) and S2 (green); and S2' (lime green). Bottom: domain architecture - the signal peptide (SP, white); the S1 subunit contains the N-terminal domain (NTD, red) and the receptor binding domain (RBD, royal blue), with the receptor binding motif (RBM, cyan); subdomain 1 (C1, yellow), subdomain 2 (C2, orange), the S2 subunit, composed successively of a fusion peptide (FP, violet) domain, heptapeptide repeat sequence 1 (HR1, lime green), HR2 (light green), central helix (CH, burgundy), the transmembrane (TM, medium sea green) domain, and the cytoplasmic domain (CT). (B) Structural mapping of spike subunits (left) and domains (right) on the full monomer model (CHARMM-GUI - COVID-19 proteins library - (Fully-glycosylated S protein head-only models (residue 1-1146) - 6VXX). A dotted blue line indicates the boundary between head (residues 27–1147) and tail. Most experimentally determined spike structures include only the head. (C) Superposition of two experimental spike structures with distinct RBD orientations: closed ('down', 6VXX, green) and open ('up', 6ZDH, red).

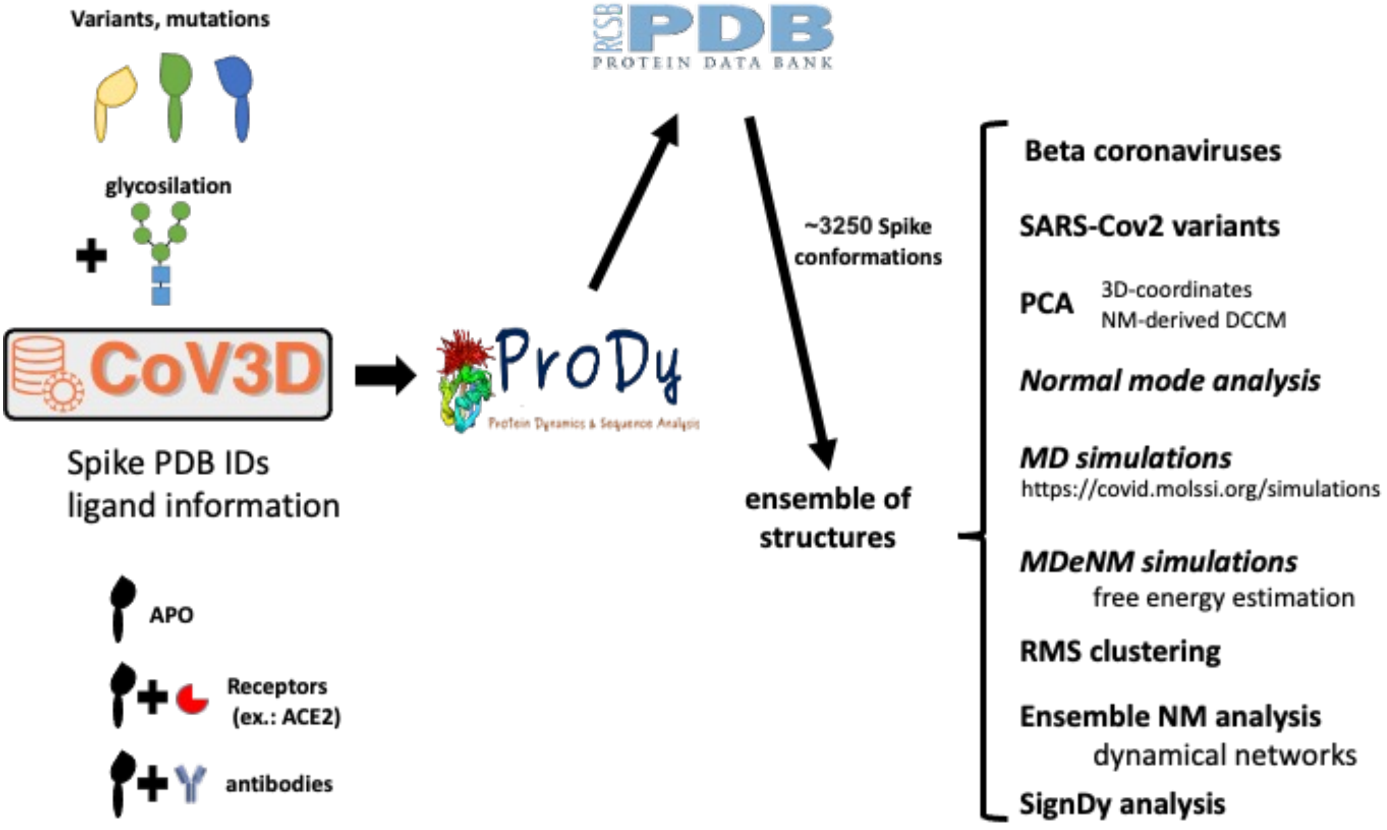

Figure S2. Pipeline for building and analyzing HCoV Spike protein ensembles. Spike protein PDB entries were obtained from Cov3D or directly from the PDB when annotations were unavailable. Cov3D provided metadata such as ligand binding and variant classification. Ensembles were built in ProDy, yielding ~3000 spike protomers. Downstream analyses included PCA, normal mode analysis (single-structure and ensemble), ligand contact mapping, variant classification, inter-species comparisons, standard and hybrid MD simulations, clustering, and dynamical network analysis to characterize spike plasticity and conformational landscapes.

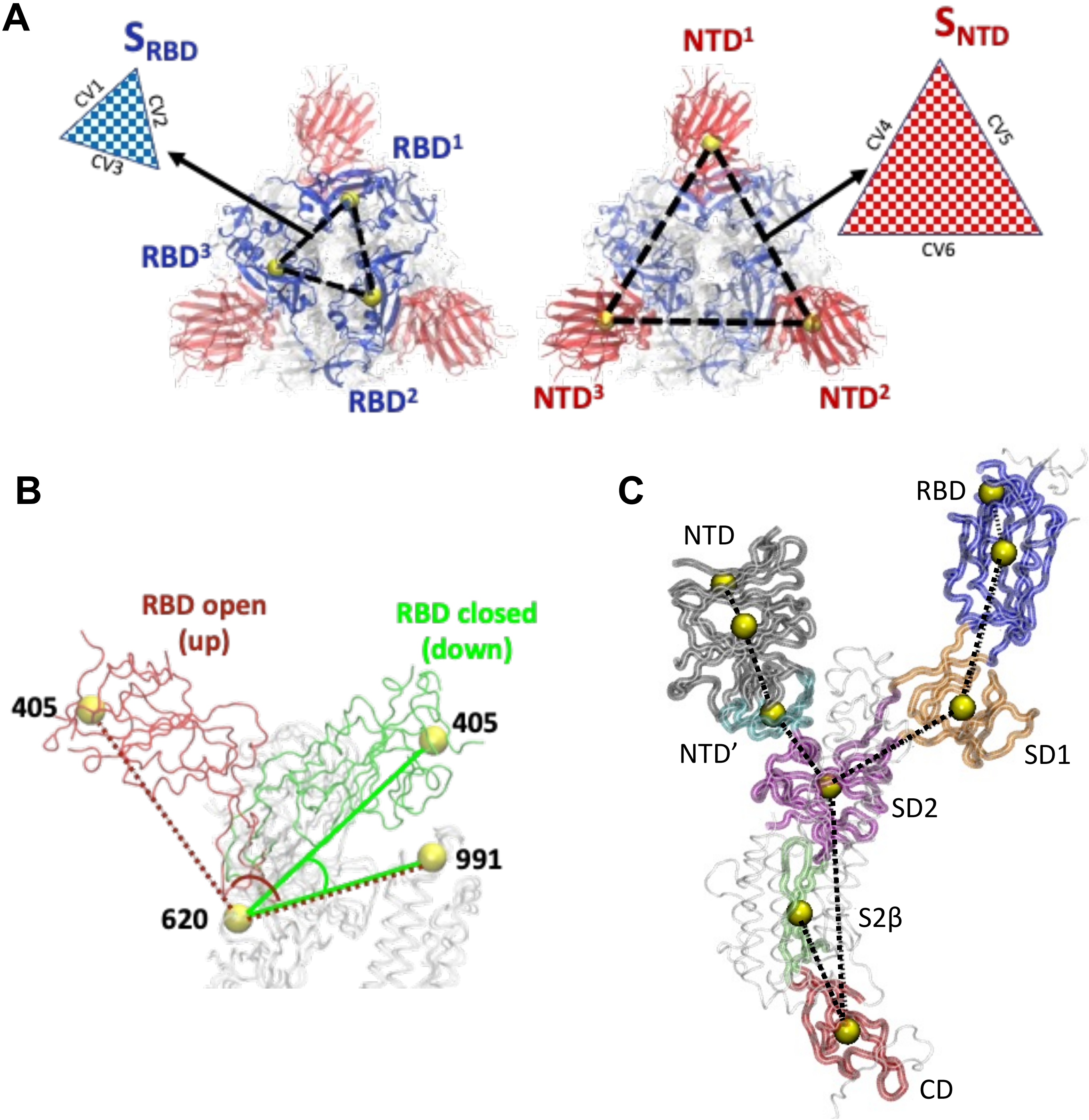

Figure S3. Key collective variables depicting spike conformational plasticity. (A,B) Top view of the trimeric spike structure with NTDs colored in red and RBDs in blue. Yellow spheres indicate the CoMs of each domain, where collective variables (CVs) 1–6 correspond to inter-RBD or NTD distances, and the blue and red triangle represents the area formed by their centers. (B) RBD angle formed by residues 405–620–991, highlighting the difference between closed (green) and open (red) monomeric structures. (C) Vector-based analysis using nine points (yellow) mapped onto the monomeric spike structure, with corresponding structural regions labeled.

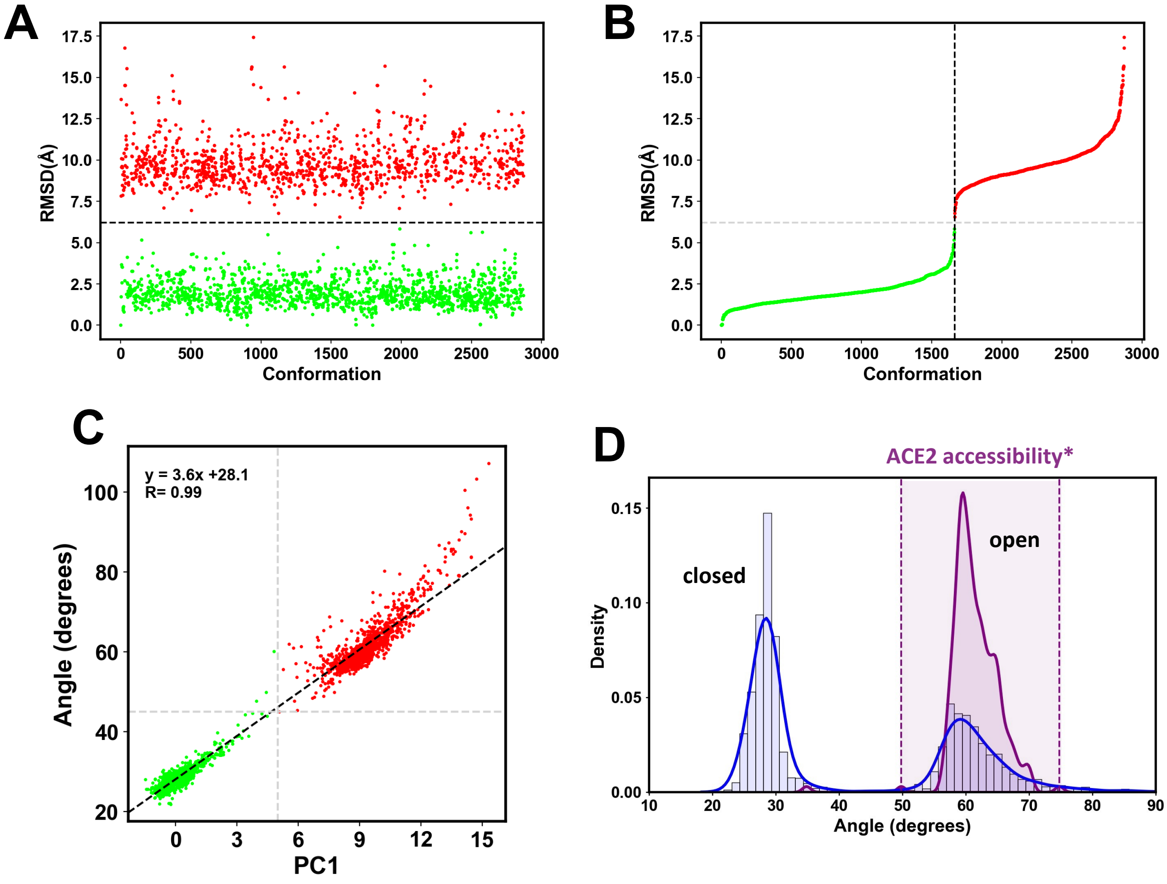

Figure S4. Spike RBD conformational states mapped by global structural descriptors. (A–C) RMSD values of ensemble structures relative to the reference (6VXX, chain A): (A) RMSD values colored by RBD conformation—green (closed) and red (open); (B) RMSD values sorted in ascending order; (C) Correlation between the RBD angle and PC1 projections. (D) Distribution of RBD angle values, with the ACE2-accessible range shaded in purple. *A mink spike structure bound to a closed RBD conformation is excluded from the accessibility range.

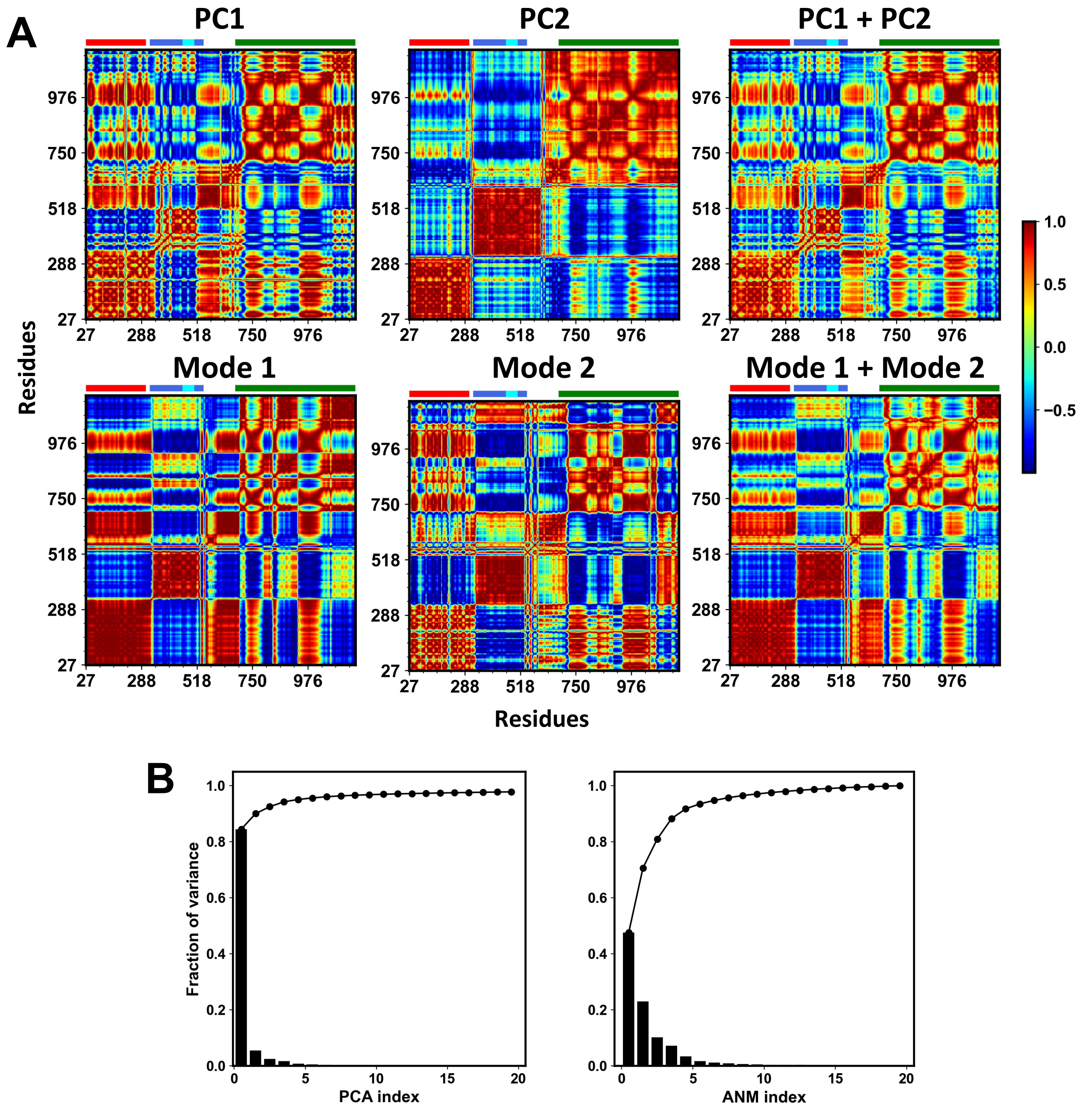

C

	Mode 1	Modes 1-2	Modes 1-3	Modes 1-4	Modes 1-5
PC1	0.37	0.81	0.86	0.9	0.9
PC2	0.68	0.73	0.73	0.81	0.86
PC3	0.11	0.15	0.22	0.47	0.48
PC4	0.36	0.51	0.58	0.64	0.74
PC5	0.23	0.23	0.24	0.29	0.42

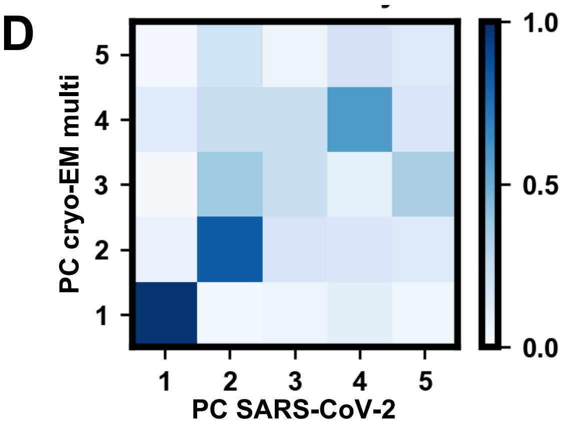

Figure S5. Close correspondence between the experimental PCs and normal modes of the spike protein. (A) Cross-correlation matrices for PC1, PC2, Mode 1, and Mode 2, shown individually and cumulatively (PC1+2, Mode 1+2). (B) Variance explained by each PC (left) and normal mode (right), with bars representing individual contributions and dots indicating cumulative variance. (C) Subspace overlap matrix quantifying the correlation between PCs and normal modes, colored on a blue scale. (D) Overlap between experimental SARS-CoV-2 ensemble PCs and those from the cryo-EM multi-state model of the Beta variant spike, also shown on the blue scale.

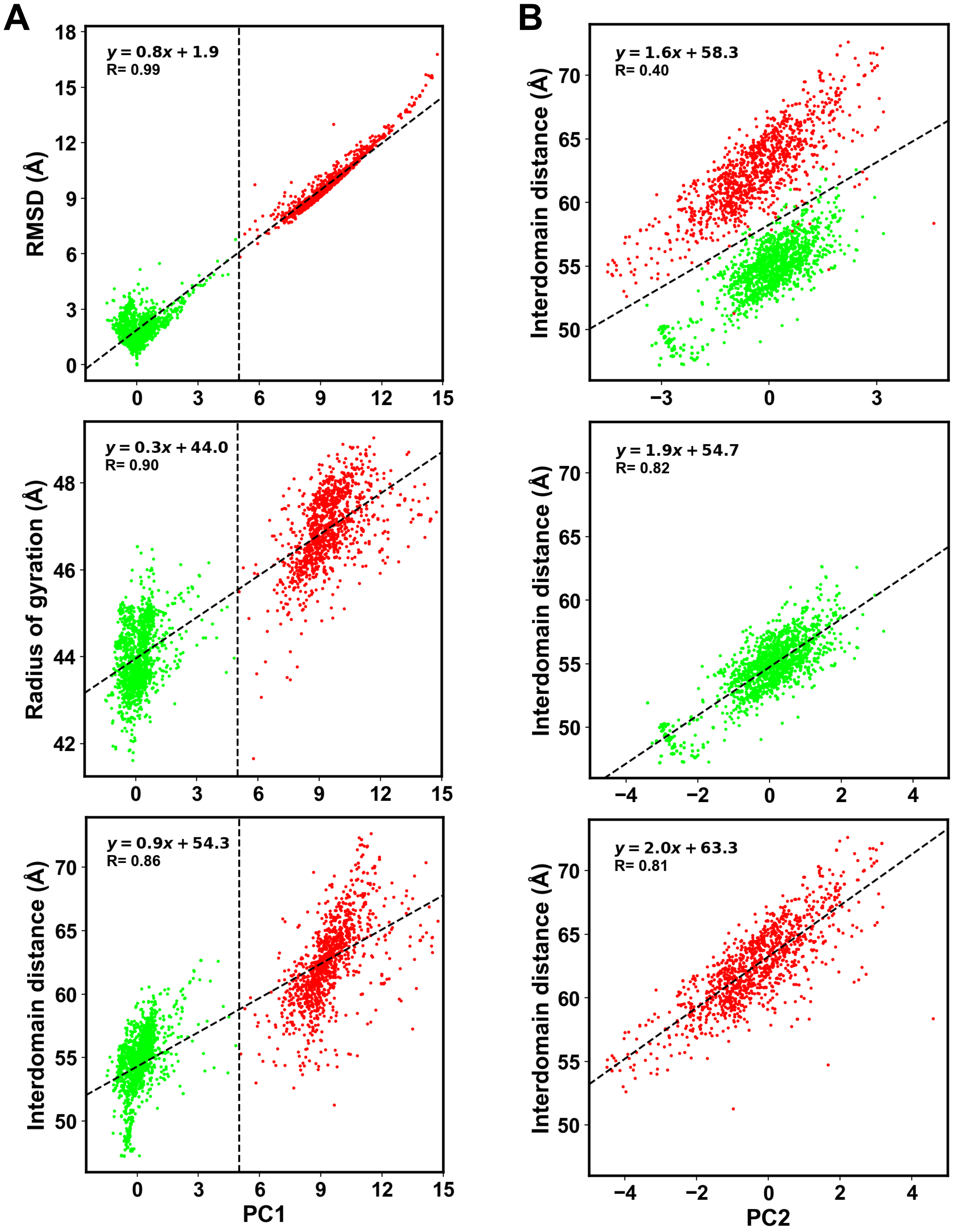

Figure S6. Global analyses of the SARS-CoV-2 experimental ensemble. (A, B) Correlations between structural descriptors and principal component projections. (A) RMSD, radius of gyration and NTD/RBD interdomain distances plotted against PC1 values. (B) Interdomain distances plotted against PC2 values for all ensemble structures (top), open RBD conformations (middle), and closed RBD conformations (bottom). Linear regression lines are shown, with corresponding equations and Pearson correlation coefficients (R) indicated in the top left corner of each plot.

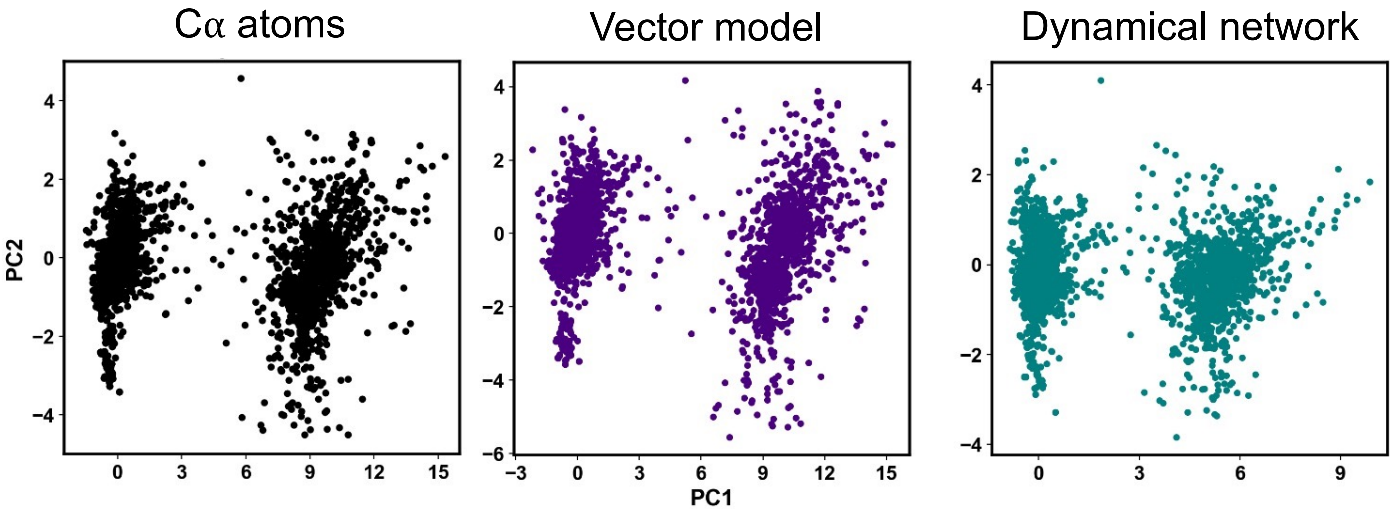

Figure S7. Comparison of PCs across models. Projection of three models derived from the SARS-CoV-2 spike experimental ensemble onto the PC1–PC2 space: the original model using all Cα atoms (black), a vector-based coarse-grained model with 9 atoms (purple), and a network-based coarse-grained model with 13 atoms (teal).

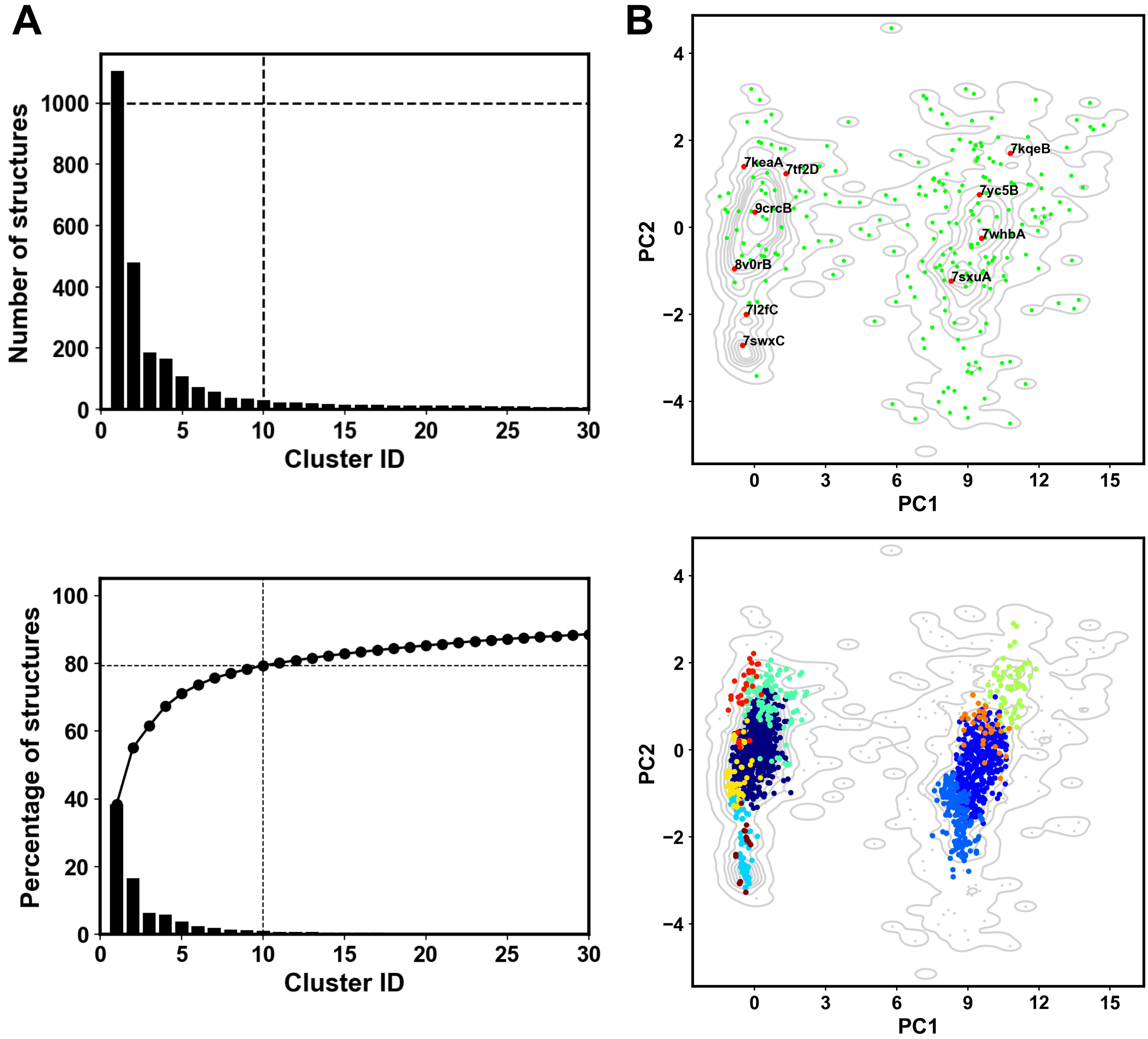

Figure S8. Clustering analysis of the SARS-CoV-2 spike ensemble. (A) Top: number of SARS-CoV-2 spike monomer structures per cluster from the experimental ensemble. Bottom: cumulative percentage of ensemble structures captured within the first 30 clusters. The top 10 clusters, highlighted as representative, account for approximately 80% of the ensemble. (B) Top: distribution of cluster representatives in the PC1–PC2 space. All cluster representatives are shown in green, with the top 10 clusters highlighted in red and annotated by PDB ID and chain. Gray contours represent the overall distribution of the full ensemble in PC1–PC2 space. Bottom: PC1–PC2 distribution of structures belonging to each of the top 10 clusters, shown in distinct colors, overlaid on the same contour background.

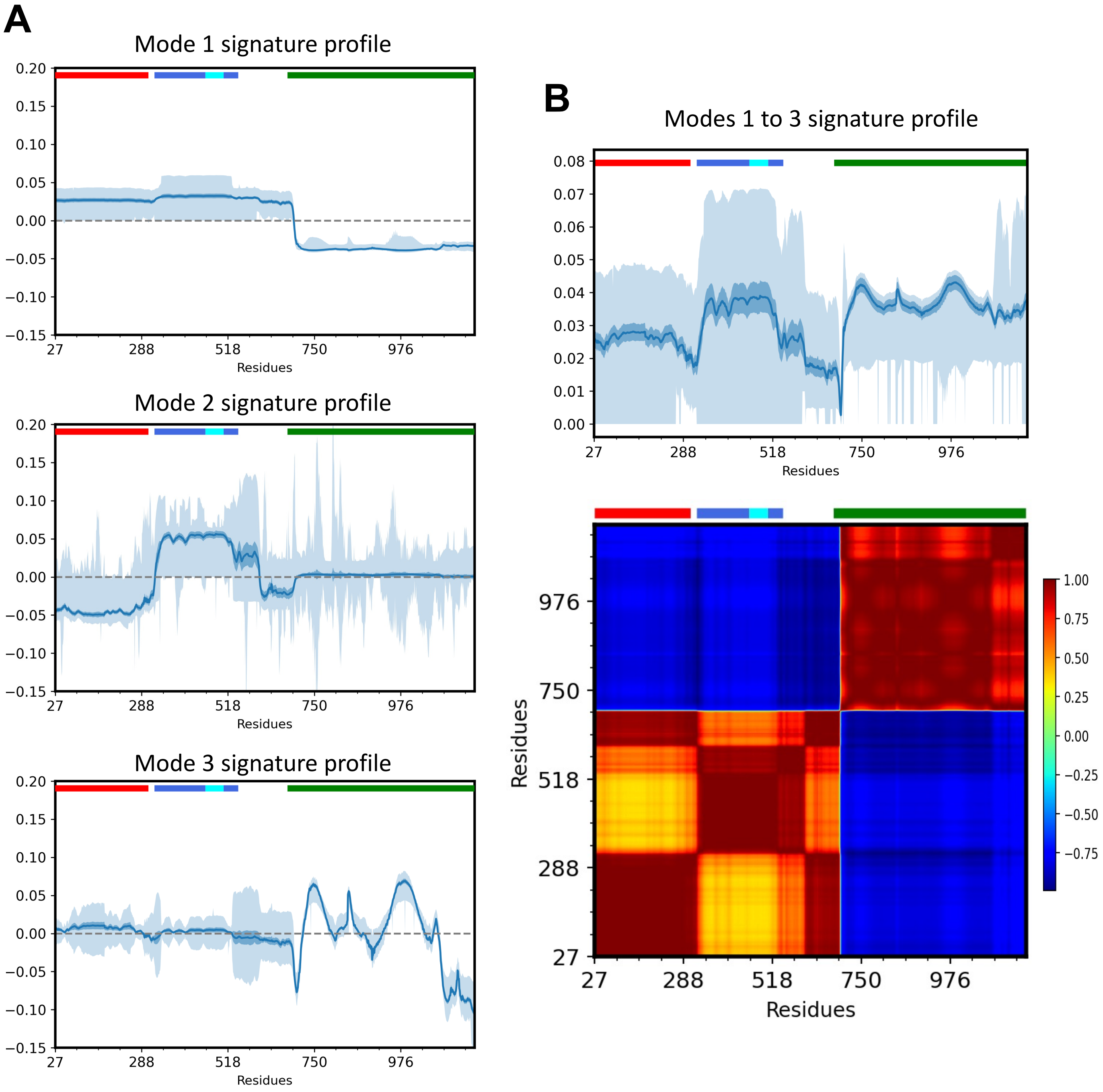

Figure S9. Signature profile of the ensemble normal modes calculated over the SARS-CoV-2 experimental ensemble. (A) Mode signature profiles for the first three anisotropic network model (ANM) modes, with a color-coded domain bar highlighting regions of functional importance: NTD (red), RBD (royal blue), RBM (cyan), and S2 (green). (B) Top: cumulative mode signature plot summarizing the contribution of each residue across the modes. Bottom: cross-correlation matrix showing dynamic couplings between residues, with correlated motions in red and anticorrelated motions in blue, scaled using a jet color map.

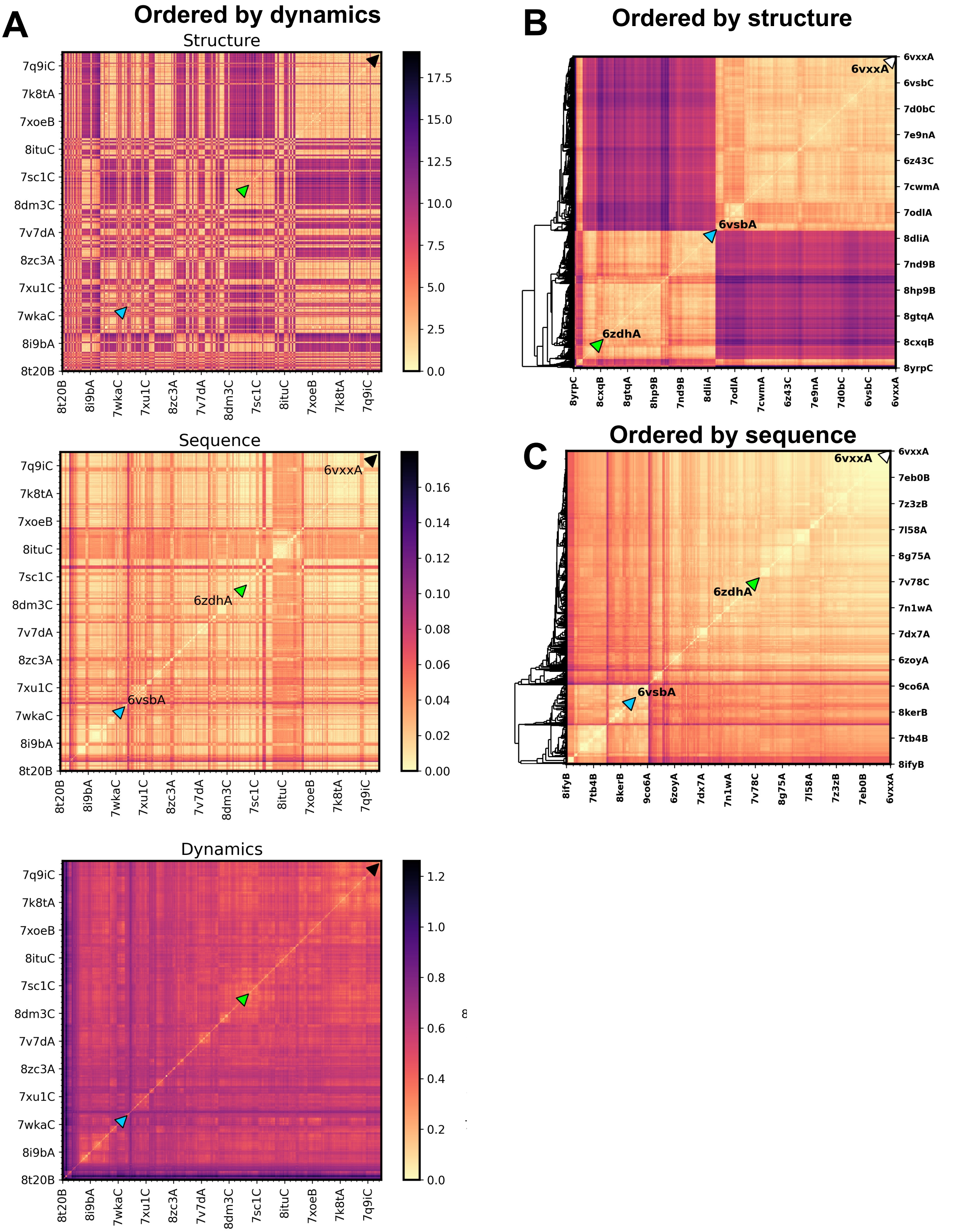

Figure S10. Signature dynamical analysis of the SARS-CoV-2 spike ensemble. (A) Comparative matrices displaying pairwise distances for structure (RMSD), sequence, and dynamics (spectral overlap of ensemble modes), all ordered by the spectral overlap matrix to enable direct comparison. Data correspond to SARS-CoV-2 spike monomers from the experimental ensemble. (B) RMSD pairwise distance matrix ordered by RMSD values, with an accompanying phylogenetic tree (left) based on structural similarity. (C) Sequence distance matrix ordered by sequence similarity, with the corresponding sequence-based phylogenetic tree shown on the left. All matrices use a reversed magma colormap (magma_r). Reference structures are marked as follows across all matrices: black (reference), cyan (open RBD protomer), and green (wide-open RBD protomer).

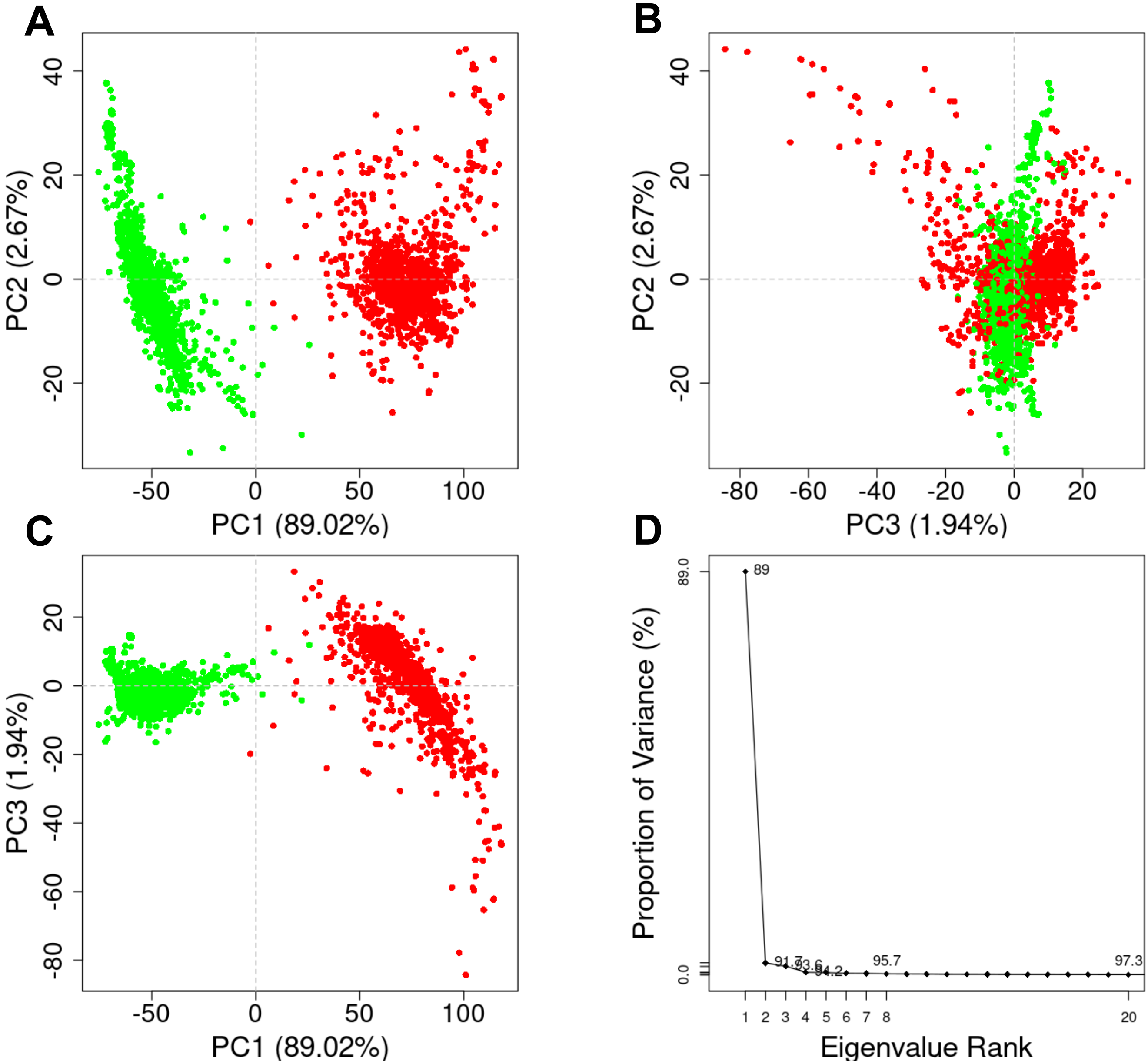

Figure S11. PCA of the DCCM derived from the SARS-CoV-2 ensemble normal modes. (A–C) Projections of the DCCM onto the PC1–PC2 (A), PC2–PC3 (B), and PC1–PC3 (C) planes. DCMM derived from the normal modes calculated for open (red) and closed (green) ensemble structures are colored according to RBD conformation. (D) The cumulative variance explained by PCs. Individual PC contributions are annotated next to each point.

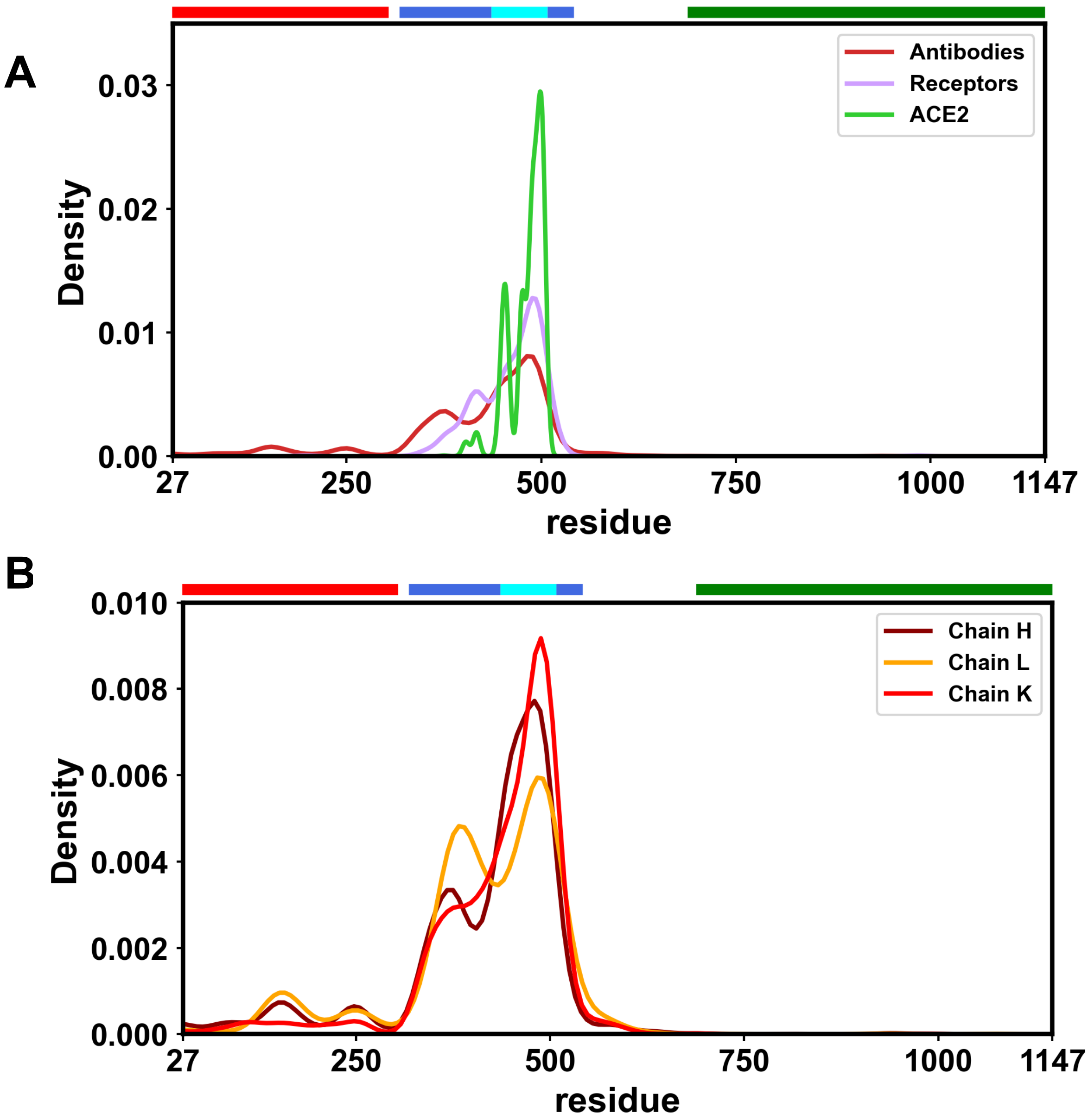

Figure S12. Residue contacts with ligands in the SARS-CoV-2 spike ensemble. (A) Density of residues located within 4 Å of ligands (including antibodies, receptors, and ACE2) across the experimental ensemble of SARS-CoV-2 spike monomers, mapped along the spike sequence. (B) Subset analysis showing residue contacts specifically with antibody chains, grouped by heavy (H), light (L), and kappa (K) chains. A color-coded domain bar above the plots highlights structural regions: NTD (red), RBD (royal blue), RBM (cyan), and S2 (green).

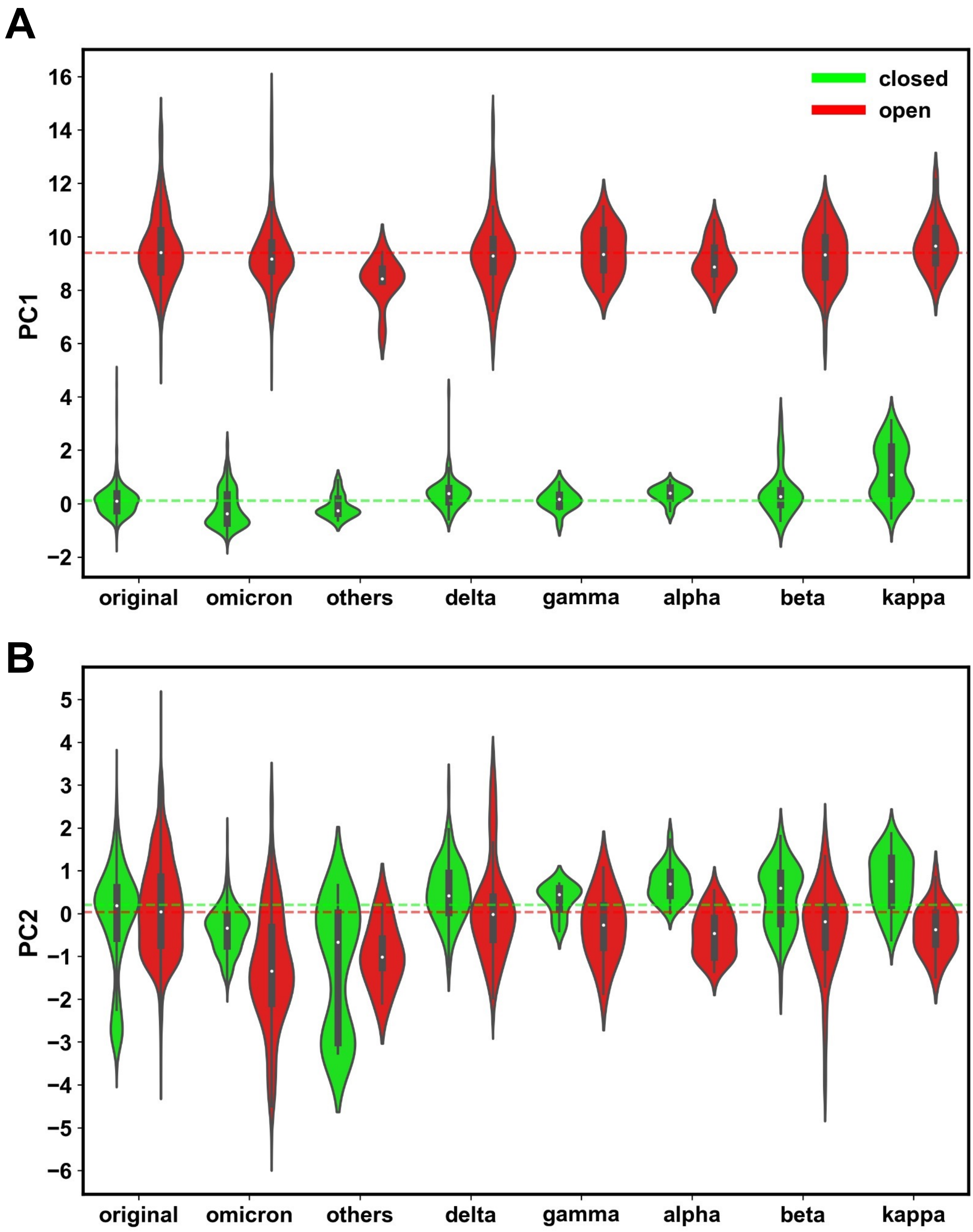

Figure S13. RBD conformation PC statistics by variant. (A, B) Violin plots showing the distribution of PC1 projection values across SARS-CoV-2 variants in the experimental ensemble of spike monomers, with RBD conformations colored as closed (green) or open (red)(A); PC2 projection distribution (B). Dotted lines indicate the median of the original group, with individual variant medians represented by their respective colors.

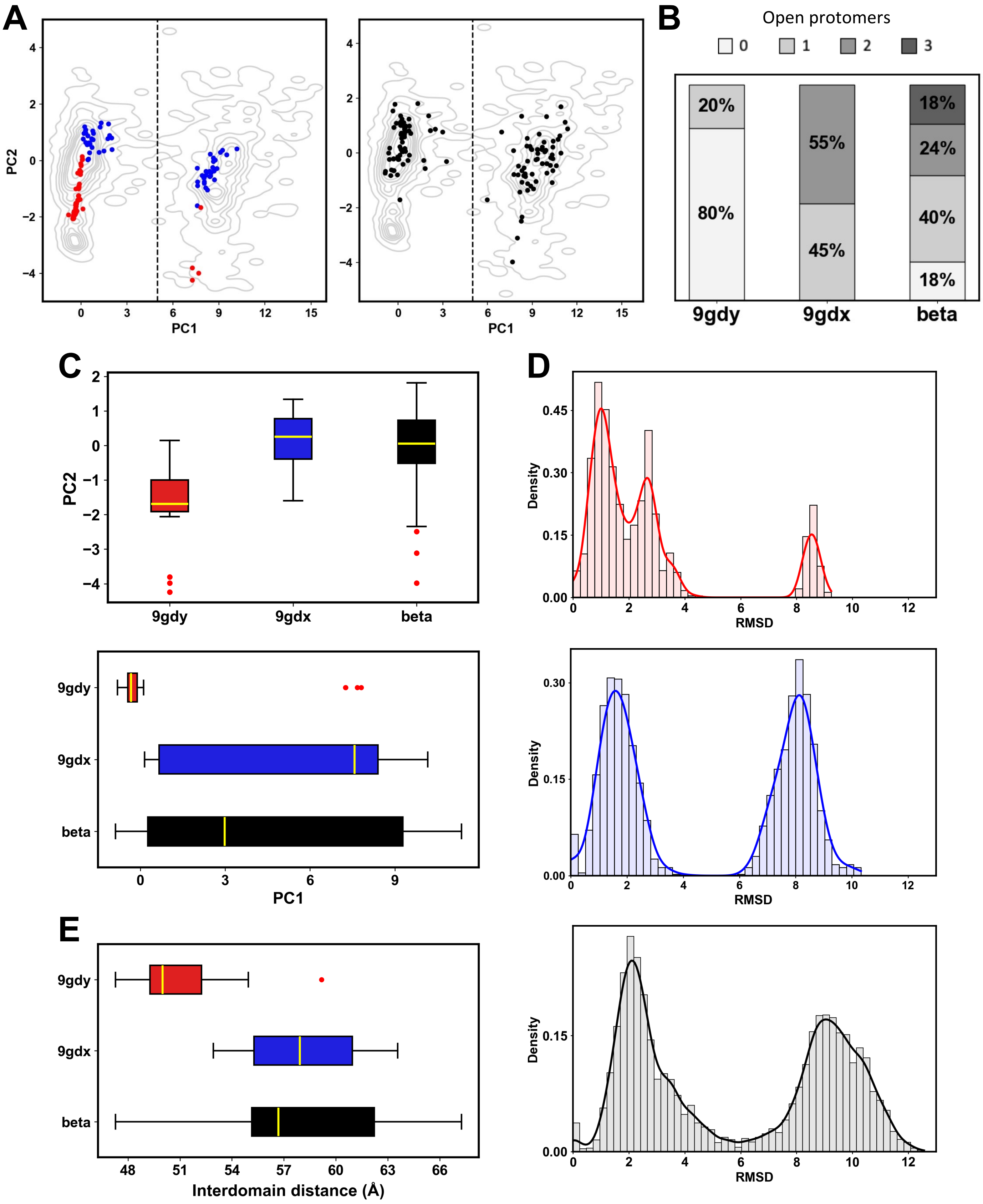

Figure S14. Multimodel cryo-EM structure analysis of SARS-CoV-2 spike. (A) PCA projection onto the PC1–PC2 space of multimodel beta spike structures 9GDX (blue) and 9GDY (red), compared to beta variant structures (black) from the experimental ensemble. (B) Distribution of the number of open protomers in the structures from (A), shown in grayscale. (C) Boxplots of PC1 (top) and PC2 (bottom) projection values, grouped and colored as in (A). (D) Density plots of pairwise RMSD values comparing multimodel cryo-EM structures and beta ensemble structures: 9GDY (red, top), 9GDX (blue, middle), and ensemble beta structures (black, bottom). (E) Boxplots of NTD–RBD interdomain distances for each group, using the same color scheme as in (A).

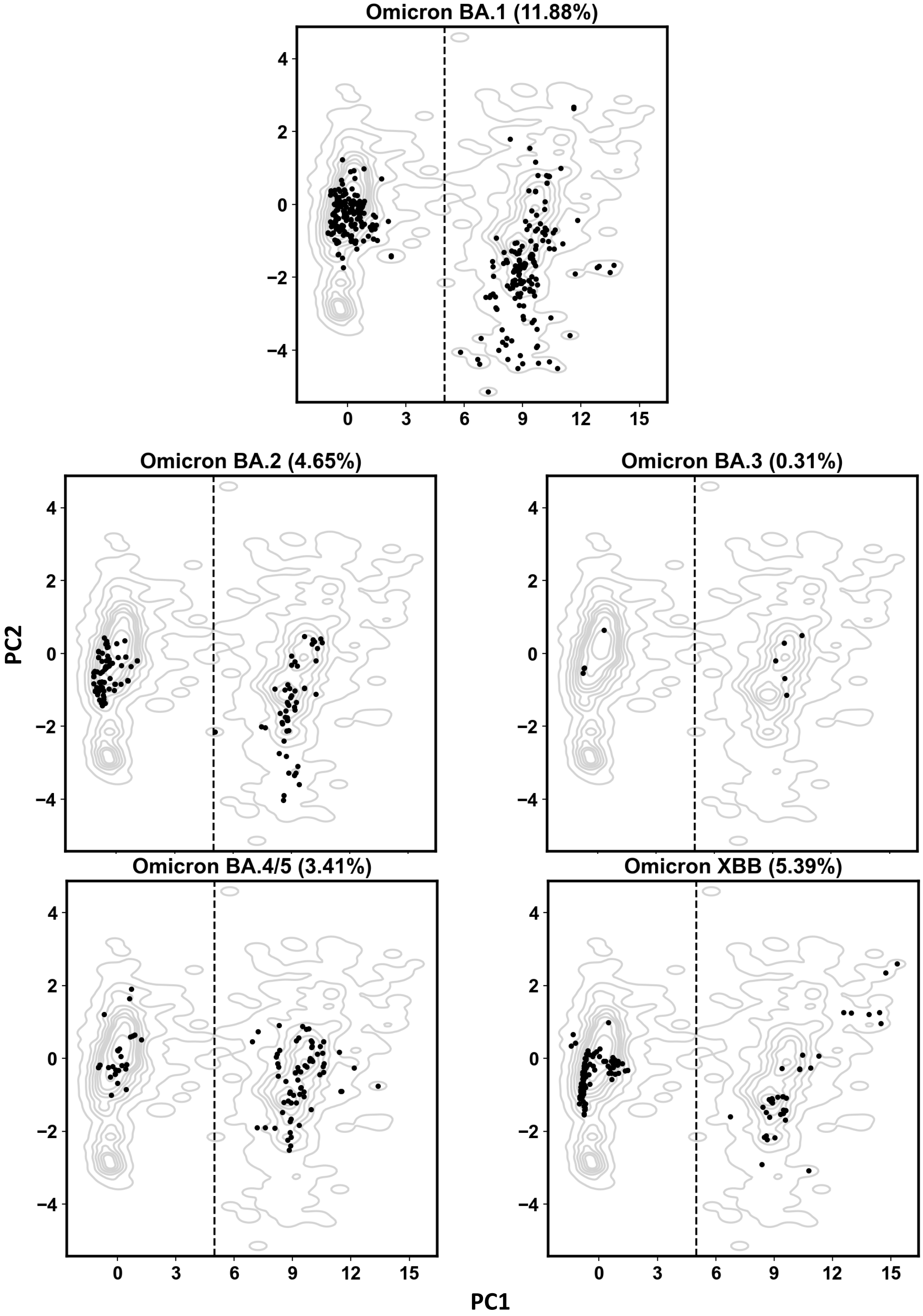

Figure S15. Contribution of Omicron subvariants to RBD conformations. Projections of the SARS-CoV-2 spike experimental ensemble, filtered by Omicron subvariant classification (*e.g.*, BA.1, BA.2, BA.4, BA.5), onto the PC1–PC2 space. The percentage of each subvariant within the total Omicron dataset is indicated. A dotted vertical line marks the boundary between closed and open RBD conformations, based on PC1 projection values (PC1 = 5). Light gray contours represent the full ensemble distribution in the PC1-PC2 space.

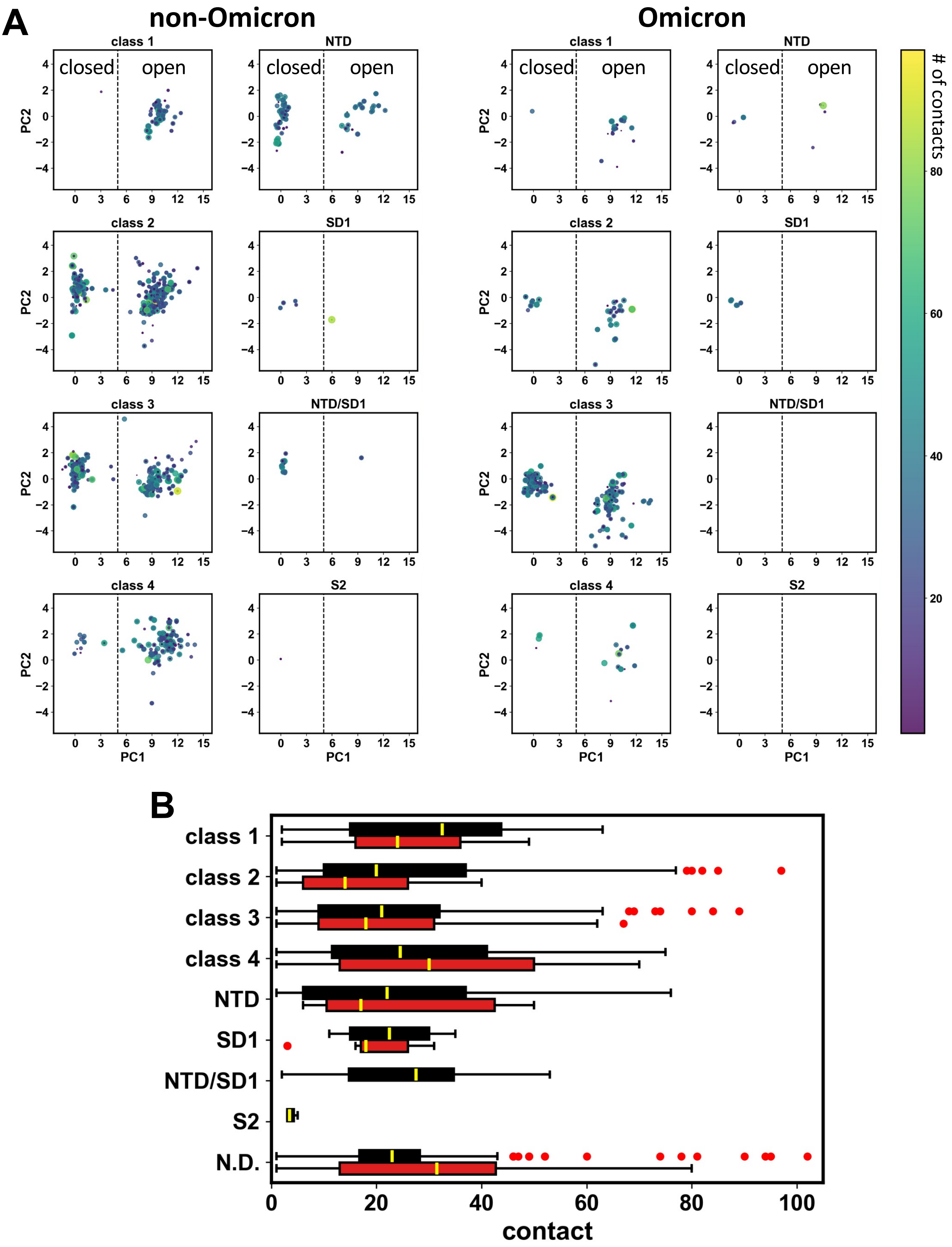

Figure S16. Interaction of different antibody classes with Omicron and non-Omicron spike variants. (A) Number of spike residues from Omicron (red) and non-Omicron (black) variants in contact (within 4 Å) with antibodies, categorized by class. Each point represents an antibody–spike complex, projected onto the first two principal components (PC1–PC2) of conformational space. Marker color and size (viridis scale) are proportional to the number of contacts. (B) Boxplots showing the distribution of contacts made by Omicron (red) and non-Omicron (black) spike residues with antibodies from different classes. Antibody classes 1 to 4 target RBD; In addition, NTD, SD1, NTD/SD1, S2 targeting antibodies are also displayed. N.D. correspond to antibodies with non-determined classes.

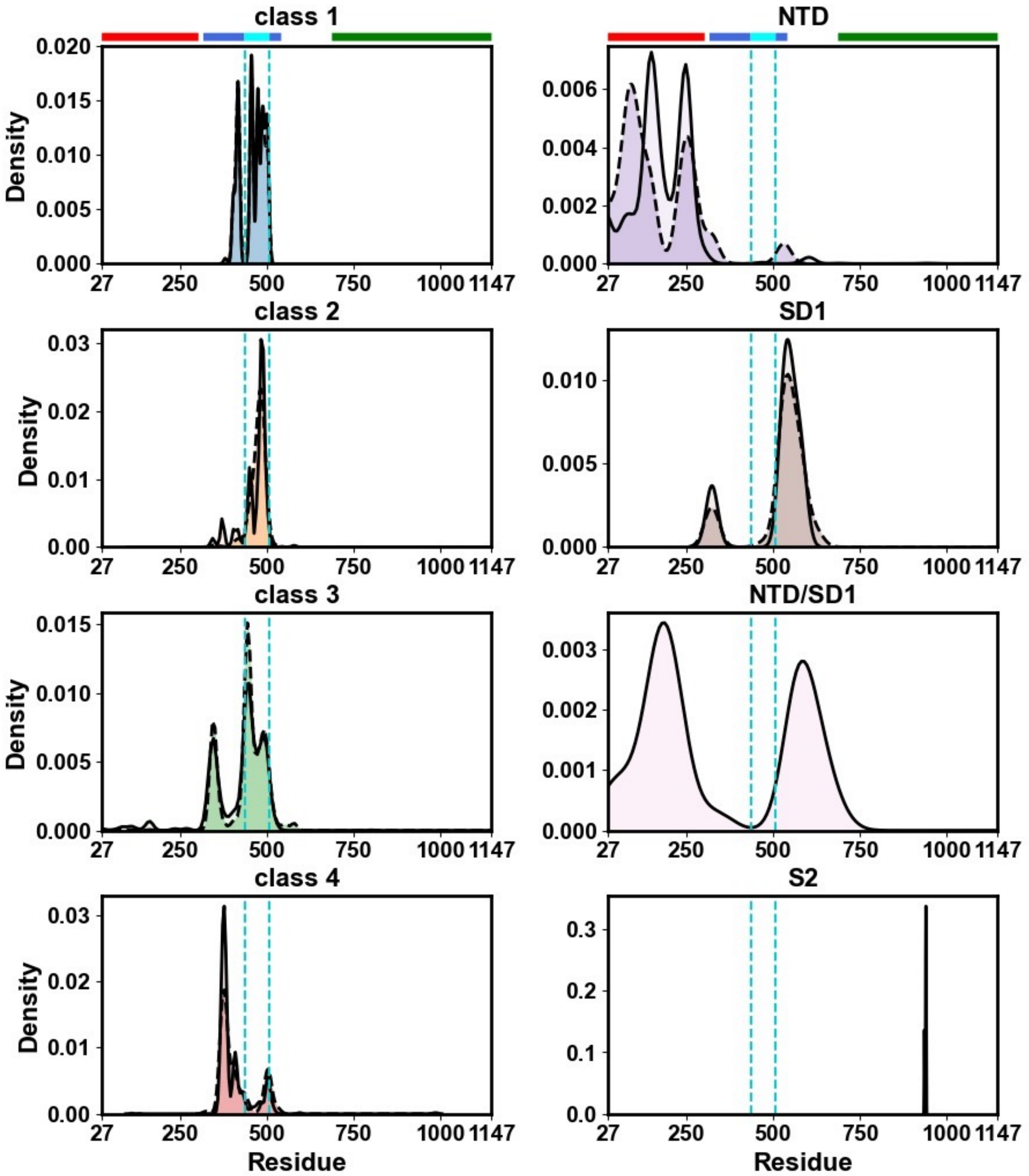

Figure S17. Distribution of Omicron and non-Omicron residue contacts with distinct antibody classes across the SARS-CoV-2 spike ensemble. Contact density profiles of Omicron (transparent fill with dashed lines) and non-Omicron (opaque fill with solid lines) residues located within 4 Å of antibody molecules, computed across an experimental ensemble of SARS-CoV-2 spike monomers and plotted along the spike sequence. A domain annotation bar above the plots denotes the locations of key structural regions: NTD (red), RBD (royal blue), RBM (cyan), and S2 sbunit (green). Antibodies are grouped by target region, including RBD-binding classes 1–4 and additional antibodies targeting the NTD, SD1, NTD/SD1, and S2 regions. Vertical cyan dashed lines corresponds to the RBM residues.
