# Supplementary figures and images for: Exploring the Intrinsic Structural Plasticity and Conformational Dynamics of Human Beta Coronavirus Spike Glycoproteins
Source: J Chem Inf Model. 2025 Jul 17;65(14):7712–33. doi: 10.1021/acs.jcim.5c00990 (PMC12308813; doi:10.1021/acs.jcim.5c00990)

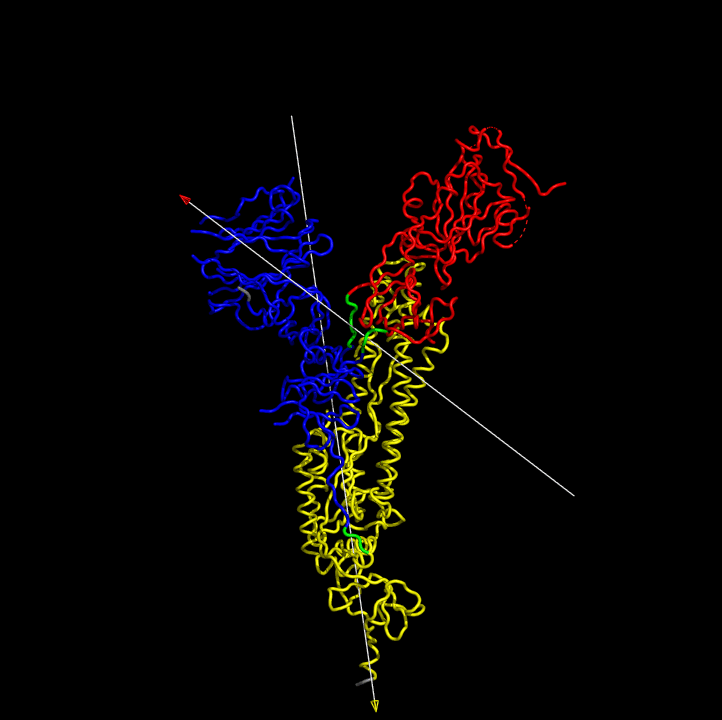

Supplement: Supplementary file 2 [file ci5c00990_si_002.zip › supp_movies/Supp_movie 8.gif]

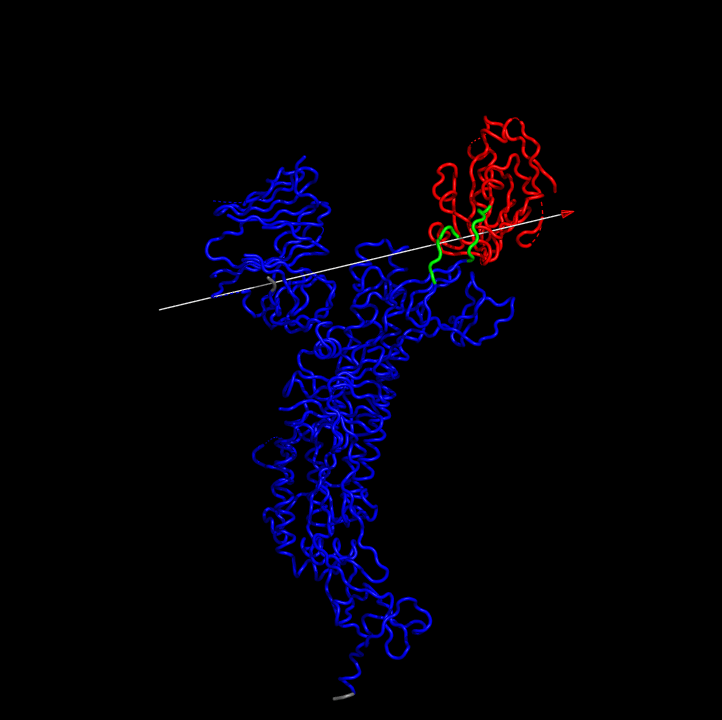

Supplement: Supplementary file 2 [file ci5c00990_si_002.zip › supp_movies/Supp_movie 9.gif]

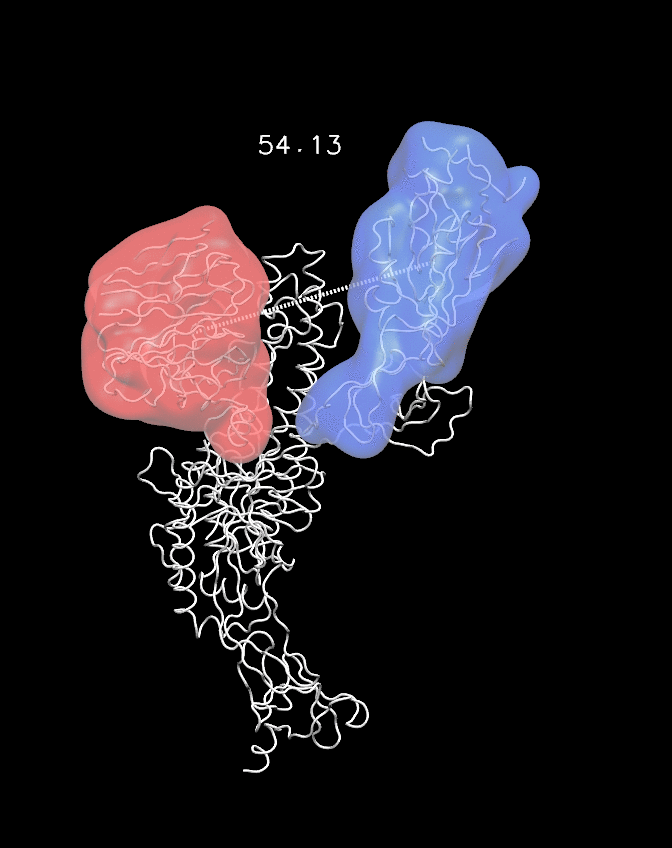

Supplement: Supplementary file 2 [file ci5c00990_si_002.zip › supp_movies/Supp_movie 1.gif]

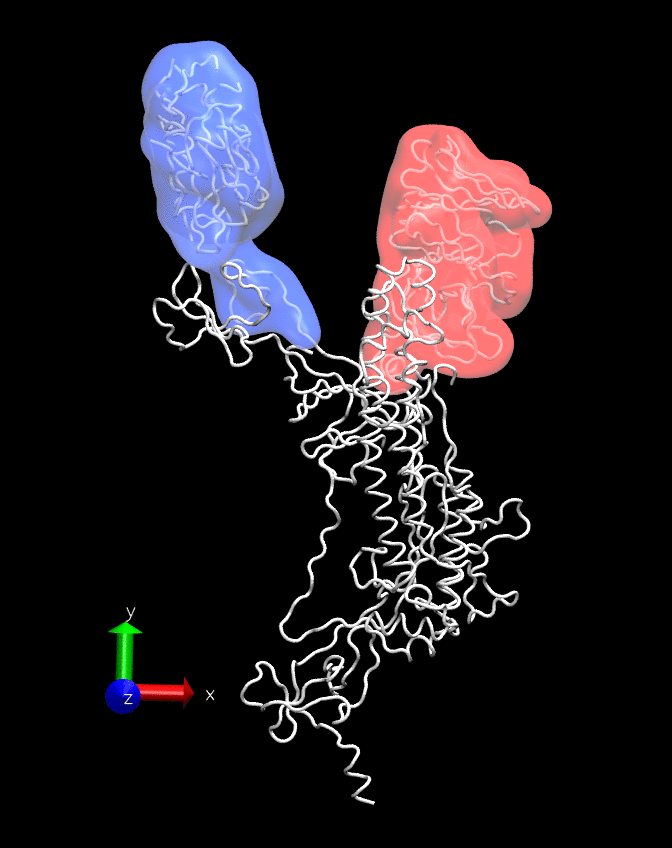

Supplement: Supplementary file 2 [file ci5c00990_si_002.zip › supp_movies/Supp_movie 2.gif]

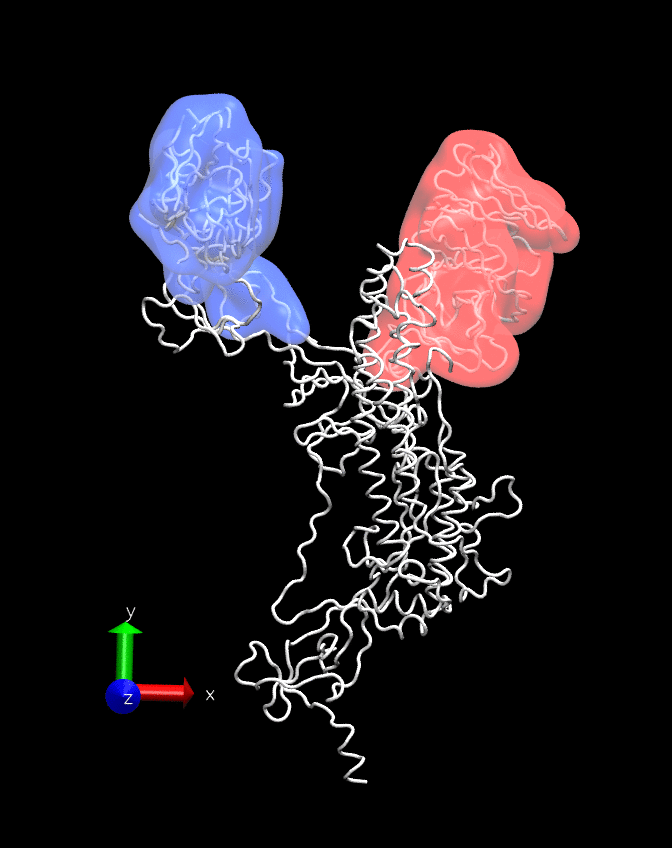

Supplement: Supplementary file 2 [file ci5c00990_si_002.zip › supp_movies/Supp_movie 3.gif]

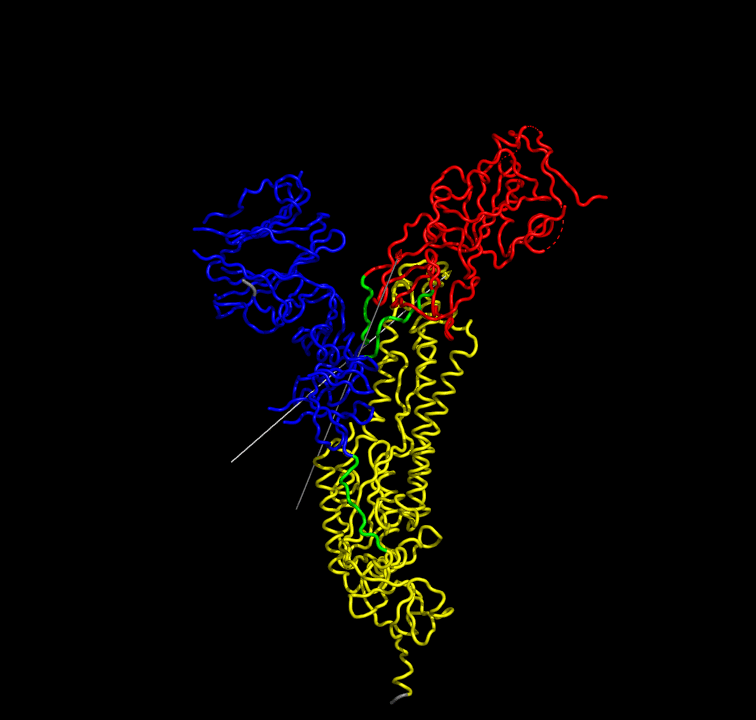

Supplement: Supplementary file 2 [file ci5c00990_si_002.zip › supp_movies/Supp_movie 7.gif]

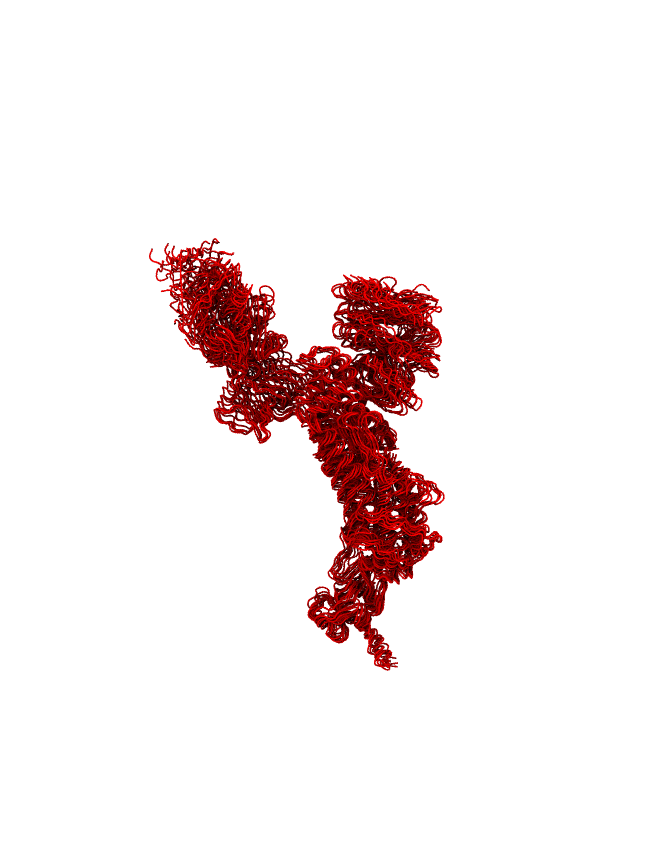

Supplement: Supplementary file 2 [file ci5c00990_si_002.zip › supp_movies/Supp_movie 6.gif]

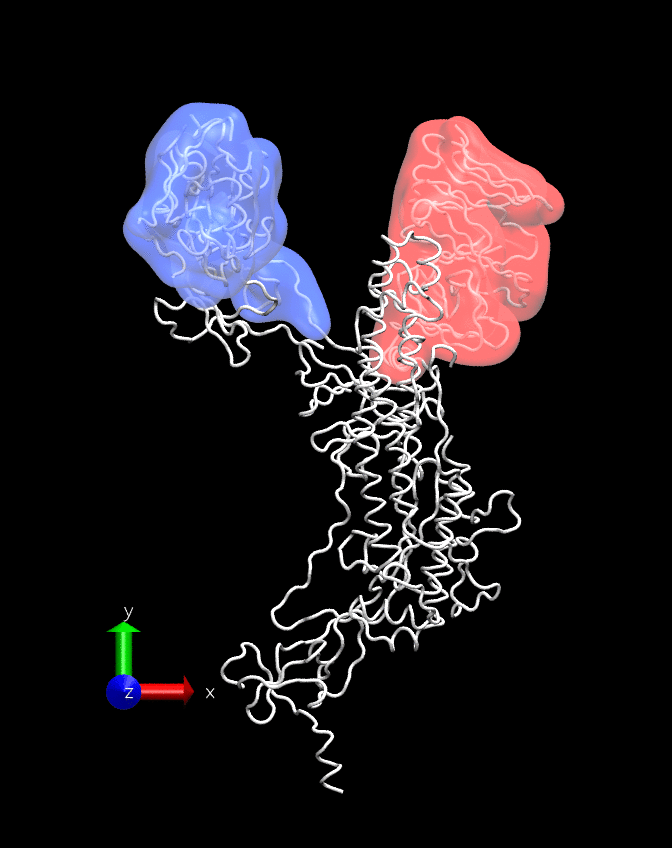

Supplement: Supplementary file 2 [file ci5c00990_si_002.zip › supp_movies/Supp_movie 4.gif]

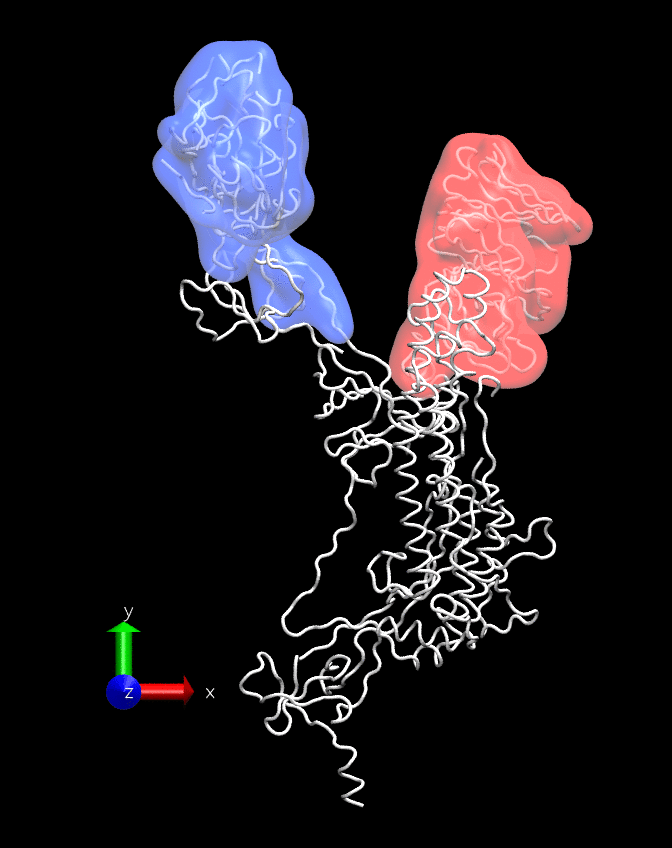

Supplement: Supplementary file 2 [file ci5c00990_si_002.zip › supp_movies/Supp_movie 5.gif]
